# Absolute lomefloxacin degradation over visible-light-driven heterojunction: experimental and theoretical insights

**DOI:** 10.1039/d6ra06407h

**Published:** 2026-10-12

**Authors:** Xinhong Gan, Qingquan Zhang, Yuqing Chen, Junyang Du, Li Sun, Jianhua Yang

**Affiliations:** a State Environmental Protection Key Laboratory of Soil Environmental Management and Pollution Control, Nanjing Institute of Environmental Science, Ministry of Ecology and Environment (MEE) of China Nanjing 210042 China; b Jiangsu Academy of Environmental Industry and Technology Corp Nanjing 210019 China; c Anhui University of Technology, School of Energy and Environment Maanshan 243002 Anhui China yangjh@ahut.edu.cn

## Abstract

Rising lomefloxacin (LMF) contamination in water raises growing health and ecological concerns, driving demand for effective removal strategies. Here we report a visible-light-responsive Fe-BiOCl/Bi_2_WO_6_ heterojunction that achieves complete degradation of LMF within 6 h under optimized conditions (pH 7.0, 5 mg per L LMF, 0.2 g per L catalyst, 500 W). Iron doping markedly enhances visible-light absorption and promotes the separation and migration of photogenerated electron–hole pairs. Mechanistic studies reveal that ˙O_2_^−^ generated at the conduction band of Fe-BiOCl, ˙OH at the valence band of Bi_2_WO_6_, and h^+^ are the primary active species, contributing approximately 76.75%, 63.22%, and 74.28% to LMF degradation, respectively. Toxicity assessment shows that the degradation products pose substantially lower risks to aquatic organisms, with reduced acute, developmental, and mutagenic effects compared to intact LMF. DFT calculations on the electronic structure and reaction pathways corroborate the experimental results, providing a mechanistic rationale for the enhanced photocatalytic performance. This work offers an efficient visible-light-driven catalyst for LMF removal and a mechanistic framework for designing doped heterojunction photocatalysts for antibiotic-contaminated water treatment.

## Abstract 2

Rising lomefloxacin (LMF) contamination in water raises growing health and ecological concerns, driving demand for effective removal strategies.

## Graphical abstract

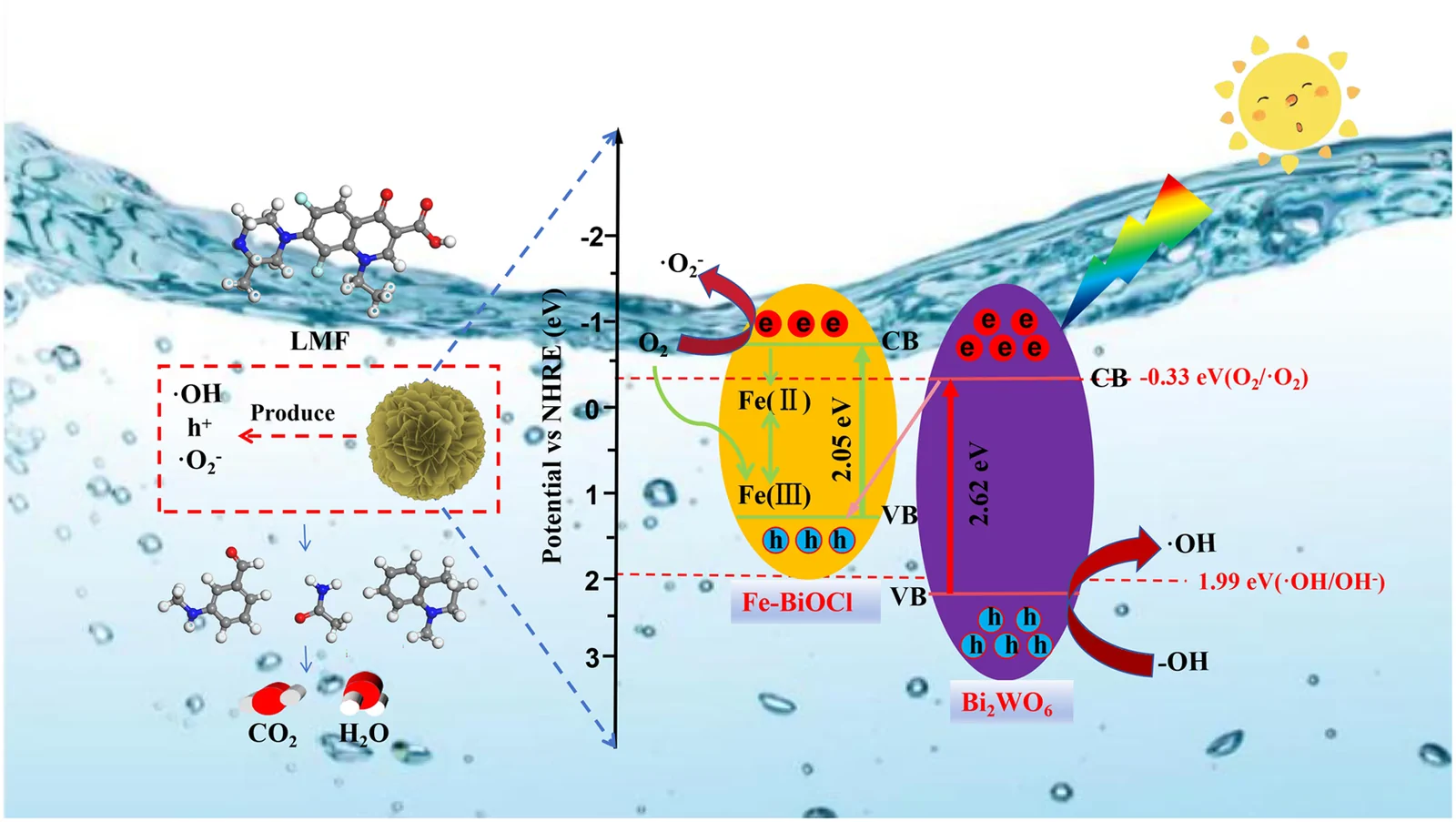

## 1 Introduction

Antibiotics are indispensable in clinical medicine and are widely used as growth promoters in livestock production.^1,2^ However, inappropriate disposal and incomplete removal have led to their release into aquatic ecosystems, progressively contaminating wastewater.^3^ Lomefloxacin (LMF), a second-generation fluoroquinolone antibiotic, is widely used in livestock and aquaculture due to its potent antibacterial activity.^4^ Its incomplete metabolism releases substantial active compounds into the environment, posing considerable ecological risks.^5^ LMF is environmentally persistent and accumulates in natural waters *via* agricultural runoff and pharmaceutical discharges.^6^ Conventional wastewater treatment plants target COD, BOD, nitrogen, phosphorus, and heavy metals, but lack processes specifically designed for antibiotics.^7^ Consequently, treated effluents often retain antibiotic residues, and traditional technologies show limited effectiveness in removing LMF.

Advanced oxidation processes (AOPs) provide an effective solution for eliminating recalcitrant organic contaminants in water. Various AOPs, including the Fenton reaction,^8^ ozonation,^9^ photocatalysis,^10^ activated persulfate,^11^ electrochemical oxidation, and ionizing radiation,^12^ have been applied to antibiotic removal. Photocatalysis, as an AOP, degrades pollutants into water and carbon dioxide^13,14^ and is regarded as a low-energy, eco-friendly technology.^15^ Therefore, developing high-performance visible-light-driven catalysts has become a major research focus for environmental remediation.^16,17^

Among various bismuth-based visible-light catalysts, BiOX (X = Br, Cl, I), belonging to the Aurivillius oxide family, possesses compatible band ordering and analogous layered structures. Their excellent electronic properties, optical performance, and stability have attracted widespread attention.^18^ Among the BiOX compounds, BiOI exhibits the optimum optical absorption and charge carrier separation capabilities, next is BiOBr and BiOCl.^19^ However, BiOI is not as stable as BiOCl and suffers from a low bandgap, which limits its ability to achieve the required redox potentials.^20^ The alternating layers of [Bi_2_O_2_]^2+^ and double halide ions provide ample space for polarization of correlative orbitals and atoms.^21^ Compared to other layered structures, the robust photocatalytic durability of BiOCl arises from the synergistic interplay between covalent bonding within its layers and van der Waals interactions across adjacent layers.^22^ Nevertheless, the catalytic function of BiOCl remains restricted on account of the inadequate energy position of the valence band (VB) maximum, which hinders effective oxidation of pollutants by photoinduced holes.^21^ To prolong carrier lifetime and enhance charge separating efficiency, various advanced strategies have been employed, like elemental doping, noble metal deposition, precise morphological control, and the construction of heterostructure with other catalysts.^23^ Among these, metal doping was recognized as an effective manner for elevating photoelectric performance.^24^ For instance, Attri *et al.*^25^ incorporated Ni into BiOCl *via* a solvothermal method. The doped material developed a prominently narrowed band gap (from 3.54 eV to 2.85 eV) and a porous framework with incremental specific surface area. The formed Ni–O bonds effectively modulated carrier recombination behavior, thereby enhancing photocatalytic activity. Similarly, Liu *et al.*^26^ prepared Co-doped BiOCl through a single-step solvothermal approach. The resulting Co-BiOCl exhibited remarkably enhanced activity, achieving 98.9% removal rate of rhodamine B with visible light in 5 minutes. Among various metal dopants, Fe doping is considered a promising modification strategy due to its unique variable valence states, which facilitate redox reactions.^27^ Therefore, this study focuses on incorporating Fe into BiOCl to develop Fe-BiOCl photocatalysts with superior photocatalytic performance.

Bismuth tungstate (Bi_2_WO_6_, BWO), one of the simplest Aurivillius phases, consists of alternating [Bi_2_O_2_]^2+^ layers and perovskite-like [WO_6_]^6−^ octahedral sheets stacked along the [001] direction.^28^ It has attracted considerable interest in the area of nanomaterials due to its favorable visible-light uptake capacity, easily synthesis, low toxicity, hybridized state formed through Bi 6s and O 2p orbitals, and excellent reactivity stability.^29^ As a typical n-type semiconductor, Bi_2_WO_6_ exhibits a bandgap ranging from 2.6 eV to 2.9 eV. Even so, its practical employment was hindered result from the quick combination of photogenerated charge carriers and its limited solar energy utilization efficiency.^30^ To alleviate such difficulties, numerous studies have pay attention to modifying Bi_2_WO_6_ through the construction of heterojunctions, which contributes to the suppression of charge recombination, leading to a superior photocatalytic activity.^31^

Herein, we propose a Z-type heterostructure formed of Fe-doped BiOCl integrated with Bi_2_WO_6_. A two-step hydrothermal approach was utilized to prepare the photocatalyst Fe-BiOCl/Bi_2_WO_6_, and it was demonstrated had highly efficient removal of lomefloxacin (LMF) through visible-light illumination with 6 hours. The constructed heterojunction effectively retards electron–hole recombination, facilitates the dissociation of electron–hole pairs, and the enlarged specific surface area affords a greater density of active sites for photocatalysis. Furthermore, Fe doping facilitates continuous electron donation and acceptance, enabling more photogenerated electrons to be transferred to O_2_, resulting in enhanced generation of ˙O_2_^−^ and accelerating the photodegradation of LMF. Electron paramagnetic resonance (EPR) spectroscopy and radical quenching tests confirmed that h^+^, ˙O_2_^−^, ˙OH and concertedly promote the oxidation of LMF into H_2_O, CO_2_ and other small molecular compounds. Herein, we present a rational design strategy for the synthesis of advanced photocatalysts that address the challenge of antibiotic contamination through efficient visible-light-driven processes.

## 2 Chemicals and methods

### 2.1 Chemicals

Lomefloxacin hydrochloride (C_17_H_20_ClF_2_N_3_O_3_, LMF, Bio-chemical grade) was purchased from Shanghai SinoChemical Reagents Company. Iron(iii) nitrate nonahydrate (Fe(NO_3_)_3_·9H_2_O), potassium chloride (KCl), sodium tungstate dihydrate (Na_2_WO_4_·2H_2_O), and bismuth(iii) nitrate pentahydrate (Bi(NO_3_)_3_·5H_2_O), all of analytical grade, were also obtained from Shanghai SinoChemical Reagents Company.

### 2.2 Catalyst fabrication

#### 2.2.1. Fe-BiOCl

Fe-BiOCl was fabricated through a one-step solvothermal approach. Fe(NO_3_)_3_·9H_2_O (0.105 mmol) and Bi(NO_3_)_3_·5H_2_O (2 mmol) were solvated in 60 mL of ethylene glycol under continuous stirring. Afterwards, to the above mixture, 2 mmol of KCl was transferred under vigorous stirring until yielding a clear and homogeneous solution. Hydrothermal synthesis was carried out by sealing the solution in a 100 mL Teflon-lined autoclave, which was then heated and maintained at 160 °C for 12 h. After cooling naturally to room temperature, the precipitate was collected and washed sequentially with absolute ethanol and deionized water. Lastly, the product was dehydrated in an baking box to acquire Fe-BiOCl. The same synthesis protocol was employed of pure BiOCl, excluding the iron source.

#### 2.2.2. Fe-BiOCl/Bi_2_WO_6_

The Fe-BiOCl/Bi_2_WO_6_ composite was fabricated through a single-step hydrothermal route. At the outset, ultrasonication was employed to solvate a mixture of Bi(NO_3_)_3_·5H_2_O (1 mmol) and Na_2_WO_4_·2H_2_O (0.5 mmol) using 10 mL of ethylene glycol as the solvent. The system was agitated for 10 minutes, with the subsequent supplementation of anhydrous ethanol, and then stirred for another 30 minutes to form a homogeneous suspension. Subsequently, 0.0388 g of freshly prepared Fe-BiOCl was dispersed into the preceding suspension under continuous whisking. A hydrothermal synthesis was conducted by sealing the mixture in a 100 mL Teflon-lined autoclave and maintaining it at 180 °C with 12 hours. The solid product was isolated after autoclave cooling and thoroughly washing with deionized ethanol and water until the supernatant was clear. Ultimately, the powder was baked and ground to acquire the Fe-BiOCl/Bi_2_WO_6_ composite. For comparison, BiOCl/Bi_2_WO_6_ was synthesized employing the same protocol by adding pure BiOCl instead of Fe-BiOCl, and pure Bi_2_WO_6_ was prepared without adding any BiOCl.

### 2.3 Characterization instrument

For detailed information on the characterization of the instruments involved in the experiment, please refer to the SI.

### 2.4 Degradation test

To assess the photocatalytic performance of catalysts, photodegradation experiments were carried out using LMF as a model compound upon exposure to visible light for 6 hours. Specifically, an aqueous solution of LMF at a fixed initial concentration was placed in a 50 mL fused silica tube, followed by the addition of a predetermined amount of the photocatalyst. An adsorption–desorption equilibrium was verified through magnetically stirring the mixture in the dark with a period of 1 hour prior to the commencement of irradiation. Thereafter, the photocatalytic reaction was initiated under simulated visible-light illumination and maintained for 6 hours. Periodic sampling (1 mL) of the suspension was conducted, after which immediate filtration with a 0.22 µm organic membrane to isolate the analyte from the catalyst prior to analysis. UV-vis spectrophotometry was employed to establish the LMF level, based on the absorbance measurement at 287 nm. All trials were conducted in triplicate, and the mean values are presented. The photochemical reactor used was a ZQ-GHX-V model (Shanghai Zhengqiao Co., China), equipped with a xenon lamp emitting simulated visible light at wavelengths between 390 and 780 nm.

### 2.5 Electrochemical analysis

Photoluminescence (PL) spectra were acquired through an Edinburgh Instruments FLS1000 spectrometer. For electrochemical measurements, a standard three-electrode system was employed. The working electrode was fabricated through dispersing the photocatalyst (10 mg) in a solution composed of Nafion (10 µL) and ethanol (1 mL), after which the solution was sonicated for 30 minutes to yield a homogeneous ink. The catalyst slurry was deposited dropwise onto a clean FTO substrate. The electrode was then spin-coated using the following procedure: 10 µL of the catalyst ink was dropped onto the FTO substrate while rotating at low speed, followed by another 10 µL aliquot before closing the lid and spinning at high speed. After spin-coating, the FTO substrate was placed on a hotplate and heated at 150 °C for 10 minutes. This coating and heating process was repeated three times. Finally, any adhesive tape was removed from the support, and it underwent annealing at 350 °C for 0.5 hours.

### 2.6 DFT calculation

First-principles calculations based on density functional theory (DFT) were employed, utilizing the CASTEP module within the Materials Studio software environment. For detailed information on methods of DFT involved in this study, please refer to Text S2 in the SI.

## 3 Results and discussion

### 3.1 Catalysts performance

#### 3.1.1. Composition and structure

The composite ratio of catalyst and the doping ratio of iron are important factors affecting the photocatalytic degradation effect. As shown in the Fig. S1, the photocatalytic effect of Fe-BiOCl/Bi_2_WO_6_ is optimal when the doping amount of Fe is 5% and the composite ratio is 10%. Therefore, we chose a composite catalyst doped with 5% iron. To identify the crystalline phases present, powder XRD patterns of all samples were collected and analyzed. Fig. S2a visually confirms all the diffraction signals of pure BiOCl were markedly corresponded to a tetragonal crystal of BiOCl (PDF # 06-0249). The dominant diffraction signals observed at 2*θ* = 11.89°, 24.09°, 25.86°, 32.61°, 33.44°, and 40.90° sequentially be assigned to the (001), (002), (101), (110), (102), and (112) crystal planes, confirming the excellent phase purity of the resulting BiOCl. As presented in Fig. S2b, upon Fe doping, the diffraction pattern of Fe-BiOCl remains largely consistent with that of pure BiOCl.^32^ However, a slight shift of certain diffraction peaks toward higher angles is observed. This shift was ascribed to the replacement of Bi^3+^ (ionic size: 1.03 Å) by smaller Fe^3+^ ions (ionic radius: 0.65 Å), which reduces the lattice constants of BiOCl and leads to the detected emission peak shift as dictated by Bragg's law.^33^ For the Bi_2_WO_6_ sample (Fig. S2c), the diffraction peaks at 2*θ* = 28.34°, 32.84°, 47.17°, 55.94°, and 58.54° are matched to the (131), (002), (202), (133), and (262) planes of the orthorhombic Bi_2_WO_6_ phase (PDF # 39-0256), indicating the successful synthesis of pure Bi_2_WO_6_. In the XRD signature of the Fe-BiOCl/Bi_2_WO_6_ catalyst (Fig. S2d), the dominant diffraction signals match well with those of Bi_2_WO_6_. Additionally, characteristic peaks belonging to BiOCl are clearly observed at 2*θ* = 11.54°, 26.10°, and 40.90°, suggesting the successful fabrication of the catalyst. No diffraction peaks corresponding to additional crystalline phases were verified, demonstrating the high phase purity of Fe-BiOCl/Bi_2_WO_6_ photocatalyst.^34^

An analysis of the vibrational modes was conducted using FTIR spectroscopy, revealing the characteristic signatures of key molecular structure and functional groups in photocatalysts. It is presented in Fig. S3, the absorption signals observed at around 3431 cm^−1^ and 1625 cm^−1^ in all samples are indicative of the H–O bending and stretching vibrational modes, respectively, which are characteristic of adsorbed water molecules.^35^ The signal at 1050 cm^−1^ is ascribed to residual nitrate species are derived from the Bi(NO_3_)_3_ starting material.^36^ For Bi_2_WO_6_, BiOCl/Bi_2_WO_6_, and Fe-BiOCl/Bi_2_WO_6_ composites, the peaks located at 730 cm^−1^ and 1381 cm^−1^ are matched to the W–O stretching vibrations and W–O–W bridging modes, respectively.^37^ Additionally, the signals at 438 cm^−1^ and 560 cm^−1^ are assigned to Bi–O linkages.^38^ In the spectra of single-phase BiOCl and Fe-BiOCl, the distinct signal at 523 cm^−1^ is indicative of the elongational vibration of O–Bi linkages,^39^ while the band at 1383 cm^−1^ is linked to the asymmetric elongational vibration of Bi–Cl linkages.^40^ After Fe doping, the FTIR spectrum of Fe-BiOCl remains largely similar to that of single-phase BiOCl. The intensity in certain regions is slightly enhanced. This suggests that Fe incorporation has only a minor influence on the molecular structure of BiOCl. No distinct Fe-related peaks are observed, likely due to the low doping concentration. Moreover, the FTIR spectra of both Fe-BiOCl/Bi_2_WO_6_ and BiOCl/Bi_2_WO_6_ composites exhibit characteristic peaks corresponding to each individual component, substantiating the successful formation of the composite materials.

Electron microscopy analysis (TEM/SEM) provided detailed insights into the surface and internal structure of the Fe-BiOCl/Bi_2_WO_6_ composite as Fig. 1a and b presented, respectively, while panels c–j present the relevant elemental mapping *via* energy-dispersive X-ray spectroscopy (EDS). It is observed in Fig. 1a, composite photocatalyst consists of numerous nanoflakes that self-assemble into microspheres with a flower-like morphology and mean particle diameter of approximately 1.2 µm. The EDS elemental mappings clearly reveal the homogeneous arrangement of Fe, O, Bi, Cl and W throughout the structure, confirming the effective doping of Fe into BiOCl and the formation of a composite between Fe-BiOCl and Bi_2_WO_6_. Furthermore, analysis of the atomic-resolution TEM image confirmed the presence of distinct atomic lattice planes exhibiting *d*-spacings of 0.26 nm and 0.24 nm. By comparison with the standard crystal structures of BiOCl (PDF # 06-0249) and Bi_2_WO_6_ (PDF # 39-0256), these values can be sequentially assigned to the (111) plane of BiOCl and the (112) plane of Bi_2_WO_6_. The clearly resolved lattice fringes provide additional support for the superior construction of Fe-BiOCl/Bi_2_WO_6_ heterojunction.

**Fig. 1:**
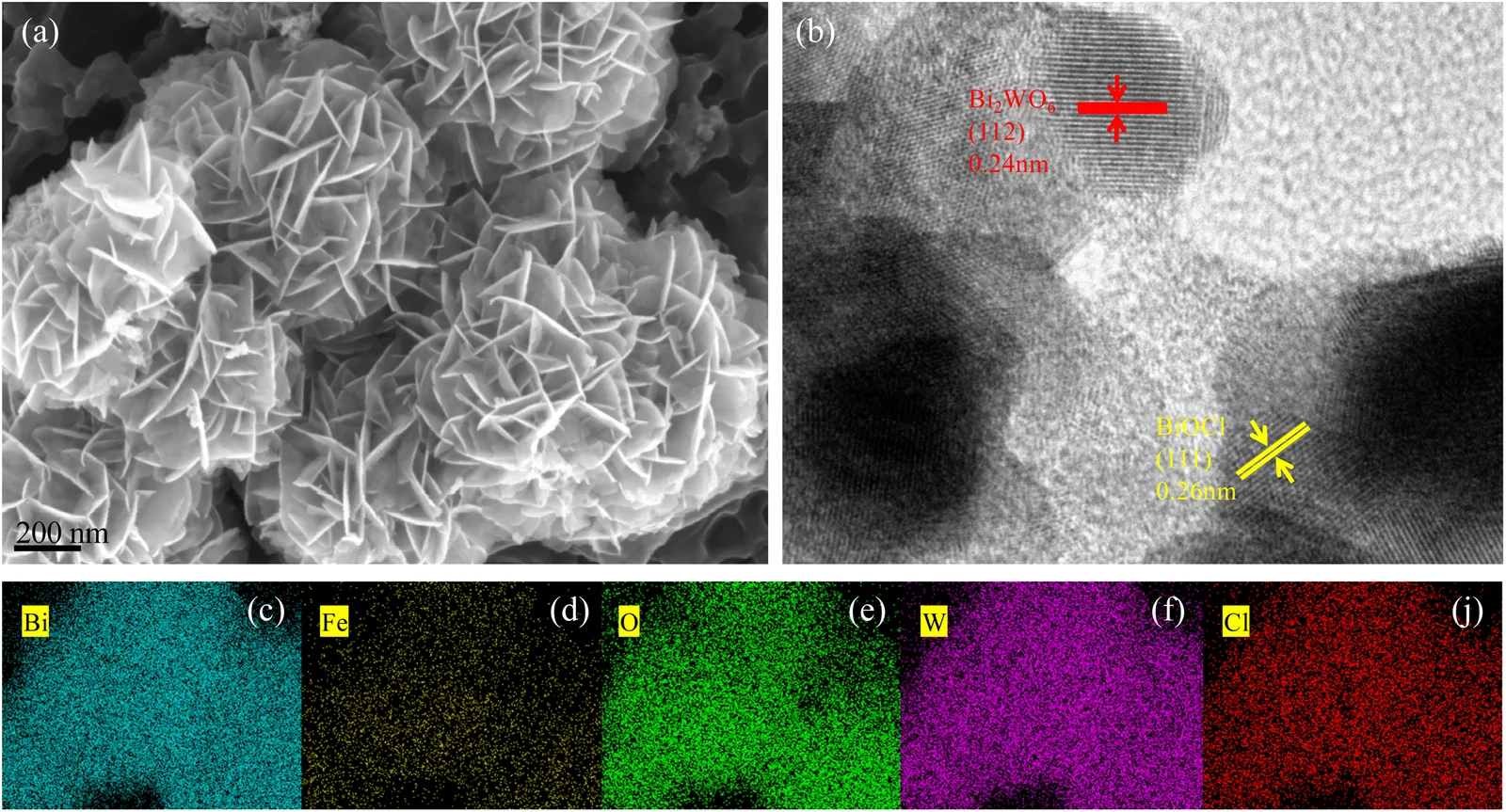
Fe-BiOCl/Bi_2_WO_6_ (a) SEM, (b) TEM and (c–j) EDS elemental mapping images of different elements.

Regarding the exploration of catalyst surfaces, in addition to observing surface morphology, we also studied the specific surface area of catalysts. The isothermal adsorption–desorption variations of materials Fe-BiOCl/Bi_2_WO_6_, Fe-BiOCl, Bi_2_WO_6_, BiOCl, and BiOCl/Bi_2_WO_6_ were analyzed. As shown in Fig. S3a, all materials follow the IV type in the Brunauer Deming Deming Teller categorization, indicating that the materials are mesoporous materials.^41^ Meanwhile, H3 hysteresis loops were observed in all samples, demonstrating the absence of narrow slit like pores in photocatalyst.^42^ The pore size, pore area and specific surface area of photocatalyst were listed in Table S1. As shown in Table S1, Fe-BiOCl (48.335 m^2^ g^−1^) exhibits a substantially larger specific surface area relative to BiOCl (31.412 m^2^ g^−1^). After composite with Bi_2_WO_6_, a significant boost in surface area was observed, evidenced by an increase from 48.335 to 65.779 m^2^ g^−1^ as measured by BET analysis, accompanied by a corresponding enhancement in pore volume from 0.114 to 0.182 cm^3^ g^−1^. The enhanced textural properties are favorable for providing abundant active sites, thereby facilitating the adsorption and subsequent degradation of LMF. Compared with BiOCl/Bi_2_WO_6_, the pore volume and surface area of photocatalyst Fe-BiOCl/Bi_2_WO_6_ have decreased to a certain extent, which may be due to the aggregation of adjacent particles induced by Fe ions and Bi_2_WO_6_.^43^ However, its specific surface area still remains at a high level, which is conducive to the progress of photocatalytic reactions. According to Fig. S4b, the pore sizes of Fe-BiOCl/Bi_2_WO_6_, Fe-BiOCl, Bi_2_WO_6_, and BiOCl/Bi_2_WO_6_ are mainly distributed in the range of 0–25 nm, while pure BiOCl has a concentrated pore size distribution range of 0–40 nm. These materials exhibit enhanced capabilities for light absorption, photon scattering, internal light reflection, and gaseous diffusion within their porous networks, thus achieving faster mass transfer and larger light collection.^44^

Specifically, the XRD results show a slight peak shift and the absence of iron-related impurity phases, directly supporting Fe incorporation into the BiOCl lattice. The TEM and HRTEM images reveal close contact between Fe-BiOCl and Bi_2_WO_6_ domains with two distinct sets of lattice fringes, providing direct evidence for the heterojunction interface. EDS mapping confirms the uniform distribution of Fe within the BiOCl domains and the coexistence of the two phases.

XPS analysis was performed to probe the elemental composition and chemical states of the material's surface. According to the scanning results, Fe-BiOCl/Bi_2_WO_6_ contains elements such as Bi, W, O, Cl, Fe, *etc.* The weak peak of Fe is owing to the low incorporation of the dopant of Fe. From the figure display, it can be seen that the signals of Bi appear at 159.1 eV and 164.4 eV, respectively, which are matched to Bi 4f_7/2_ and Bi 4f_5/2_, which are characteristics of the oxidation state of Bi^3+^ material.^49^ The XPS spectra of W 4f show signals at 35.3 eV and 37.4 eV sequentially be characteristic of W 4f_7/2_ and W 4f_5/2_, indicating that the absence of W as W^6+^ in the composite material.^50^ The Cl 2p spectrum of the Fe-BiOCl/Bi_2_WO_6_ composite (Fig. 2e) confirms the presence of Cl^−^, with its characteristic spin–orbit split peaks appearing at 198.1 eV (2p_3/2_) and 199.8 eV (2p_1/2_).^51^ The 1s spectrum of O displays two distinct signals at 530.6 eV and 529.9 eV, respectively. The O 1s signal at 530.6 eV is assigned to the lattice oxygen species and serves as a fingerprint for the Bi–O bonding environment, while the signal at 529.9 eV is consistent with oxygen in the [Bi_2_O_2_]^2+^ layer.^52^ The Fe 2p XPS spectrum is presented in Fig. 2f. The Fe 2p spectrum exhibited two peaks at 723.8 and 710.1 eV, corresponding to the Fe 2p_1/2_ and Fe 2p_3/2_ spin–orbit components.^46^ The signal at 727.3 eV match to the 2p_1/2_ orbital of Fe^3+^,^47^ whereas the signal near 730.3 eV is related to the satellite peak of Fe.^48^ Moreover, the signal at 708.76 eV pertains to Fe^2+^, which may be formed by hydrothermal reaction at high temperature and high pressure.^45^ This indicates that Fe^3+^ and Fe^2+^ in Fe doped Fe-BiOCl/Bi_2_WO_6_, combined with XRD results, suggest that Fe in the material forms strong Bi–O–Fe bonds in the BiOCl lattice through *in situ* local substitution of Bi^3+^.

**Fig. 2:**
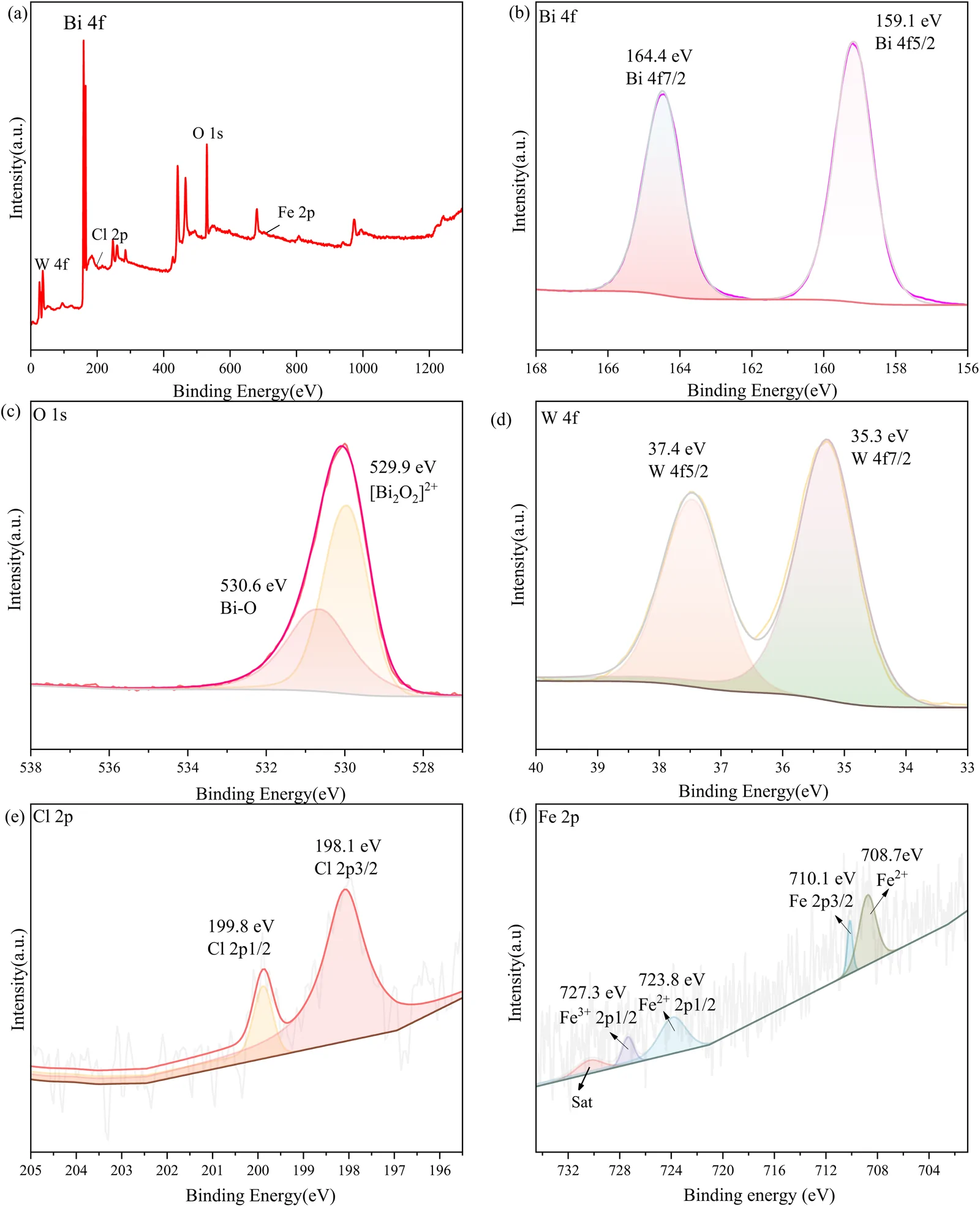
XPS of Fe-BiOCl/Bi_2_WO_6_ (a) survey of the sample, (b) Bi, (c) O, (d) W, (e) Cl, (f) Fe.

Because Fe 2p signals are complicated by multiplet splitting and satellite features, and because the binding energy ranges of Fe^2+^ and Fe^3+^ overlap, the presence of a minor fraction of Fe^2+^ cannot be fully excluded based on XPS alone. Therefore, the Fe valence state is assigned as predominantly Fe^3+^ rather than a single, definitively confirmed oxidation state. Definitive confirmation of the local Fe coordination environment would require element-specific structural probes such as EXAFS, which is beyond the scope of the present work but would be valuable for future investigation.

#### 3.1.2. Photoelectrochemical property

The light-harvesting capabilities of the powder catalysts were evaluated using UV-vis diffuse reflectance spectroscopy (DRS), as presented in Fig. 3a, which also compares their photocatalytic activities and bandgap energies. Compared to pure BiOCl, Fe-doped BiOCl exhibits enhanced light absorption and a broader visible-light response, accompanied by a reduction in bandgap. Further improvement in photocatalytic performance was achieved after constructing a composite between Fe-BiOCl and Bi_2_WO_6_. In contrast, the undoped composite, BiOCl/Bi_2_WO_6_, showed inferior light absorption relative to the Fe-BiOCl/Bi_2_WO_6_. The observed improvement arises primarily from the incorporation of Fe^3+^ ions, which possess low bandgap energy and leads to the creation of discrete defect energy levels within its band structure. The doping of Fe effectively extends the photoresponse range of the material.^53^ Based on Tauc plot analysis, the estimated bandgap values of Fe-BiOCl, Bi_2_WO_6_, Fe-BiOCl/Bi_2_WO_6_, BiOCl, and BiOCl/Bi_2_WO_6_ are sequentially 2.05 eV, 2.62 eV, 1.92 eV, 3.24 eV, and 2.36 eV.

**Fig. 3:**
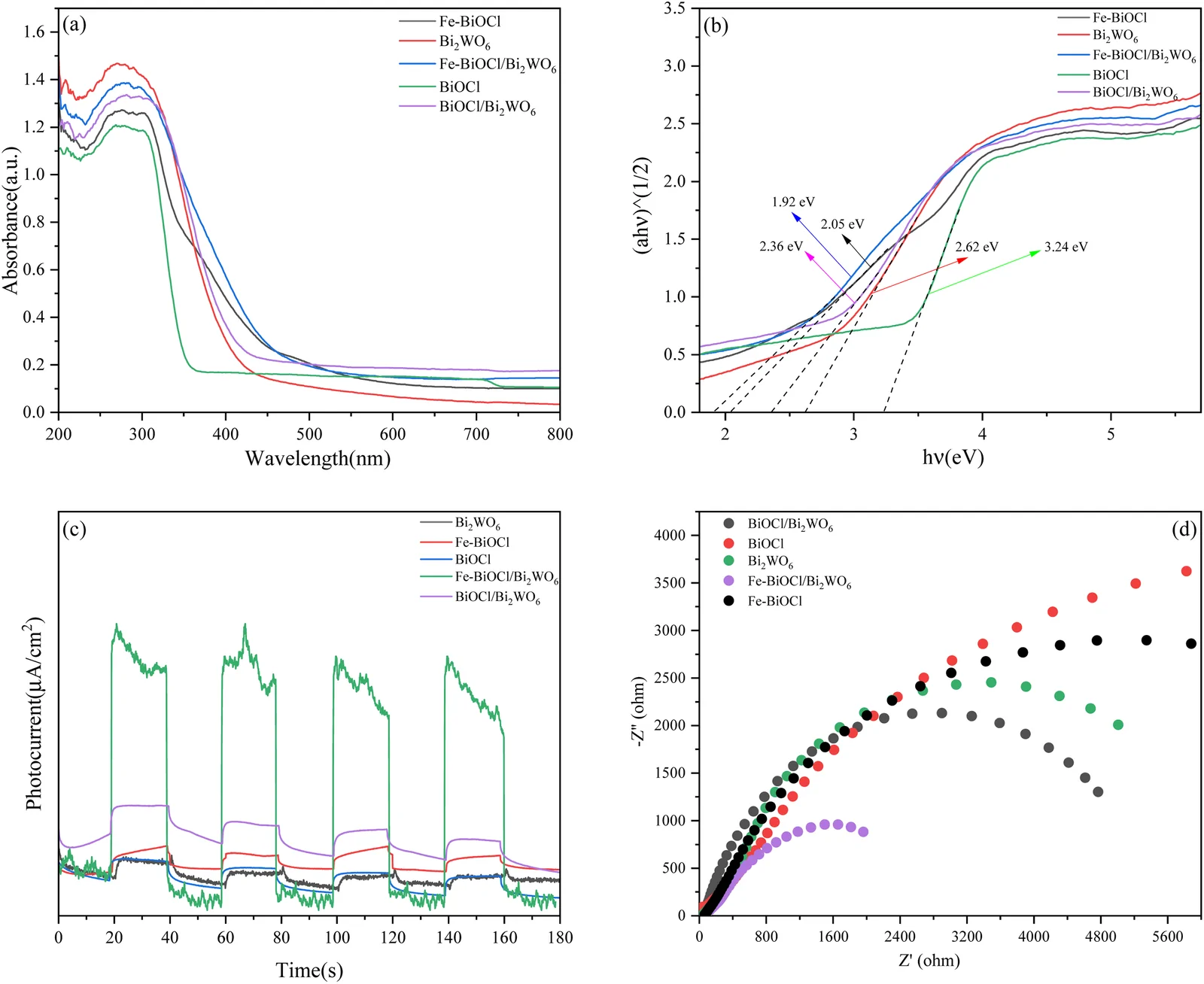
(a) UV-vis spectra, (b) bandgap calculations, (c) chronocurrent experiments and (d) electrochemical impedance diagram of BiOCl, Fe-BiOC, Bi_2_WO_6_, BiOCl/Bi_2_WO_6_ and Fe-BiOCl/Bi_2_WO_6_.

Photocatalytic activity hinges on efficient charge carrier dynamics. To elucidate the charge separation and transport properties, we conducted a combined analysis using electrochemical impedance spectroscopy (EIS) and transient photocurrent (IT).^54^Fig. 3c presents the transient photocurrent responses of Bi_2_WO_6_, BiOCl, BiOCl/Bi_2_WO_6_, Fe-BiOCl and Fe-BiOCl/Bi_2_WO_6_ when exposed to visible light. Neat Bi_2_WO_6_ and BiOCl display relatively weak photocurrent densities, whereas the composite materials exhibit enhanced and stable photocurrent responses. Notably, the Fe-BiOCl/Bi_2_WO_6_ hybrid demonstrates the highest photocurrent intensity, suggesting that both iron-doped and heterojunction construction synergistically enhances charge dissociation and suppresses bulk recombination, thereby boosting the available charges for surface reactions.

To further elucidate the charge migration behavior, EIS analysis was performed, and the corresponding Nyquist plots are displayed in Fig. 3d. Normally, the charge-transfer resistance is inversely proportional to the arc radius.^55^ The Fe-BiOCl sample shows a reduced arc radius compared to pure BiOCl, suggesting that Fe doping promotes charge carrier mobility. The Fe-BiOCl/Bi_2_WO_6_ composite exhibits the smallest arc radius. This indicates the lowest charge-transfer impedance. It also suggests the most efficient spatial separation of photogenerated charge carriers.

Photoluminescence (PL) spectroscopy was employed to assess the recombination behavior of photoinduced electron–hole pairs in the catalysts. It is shown in Fig. 4a, all composite catalysts exhibit reduced PL intensities, with the Fe-BiOCl/Bi_2_WO_6_ hybrid showing the weakest emission. This suggests that the heterojunction constructed from Fe-BiOCl and Bi_2_WO_6_ effectively suppresses the recombination of charge carriers. Furthermore, in contrast to pure BiOCl, Fe-doped BiOCl demonstrates a decrease in PL intensity, suggesting that Fe doping facilitates interfacial charge transfer and thereby enhances the photocatalytic activity. The above results are align well with the EIS data.

**Fig. 4:**
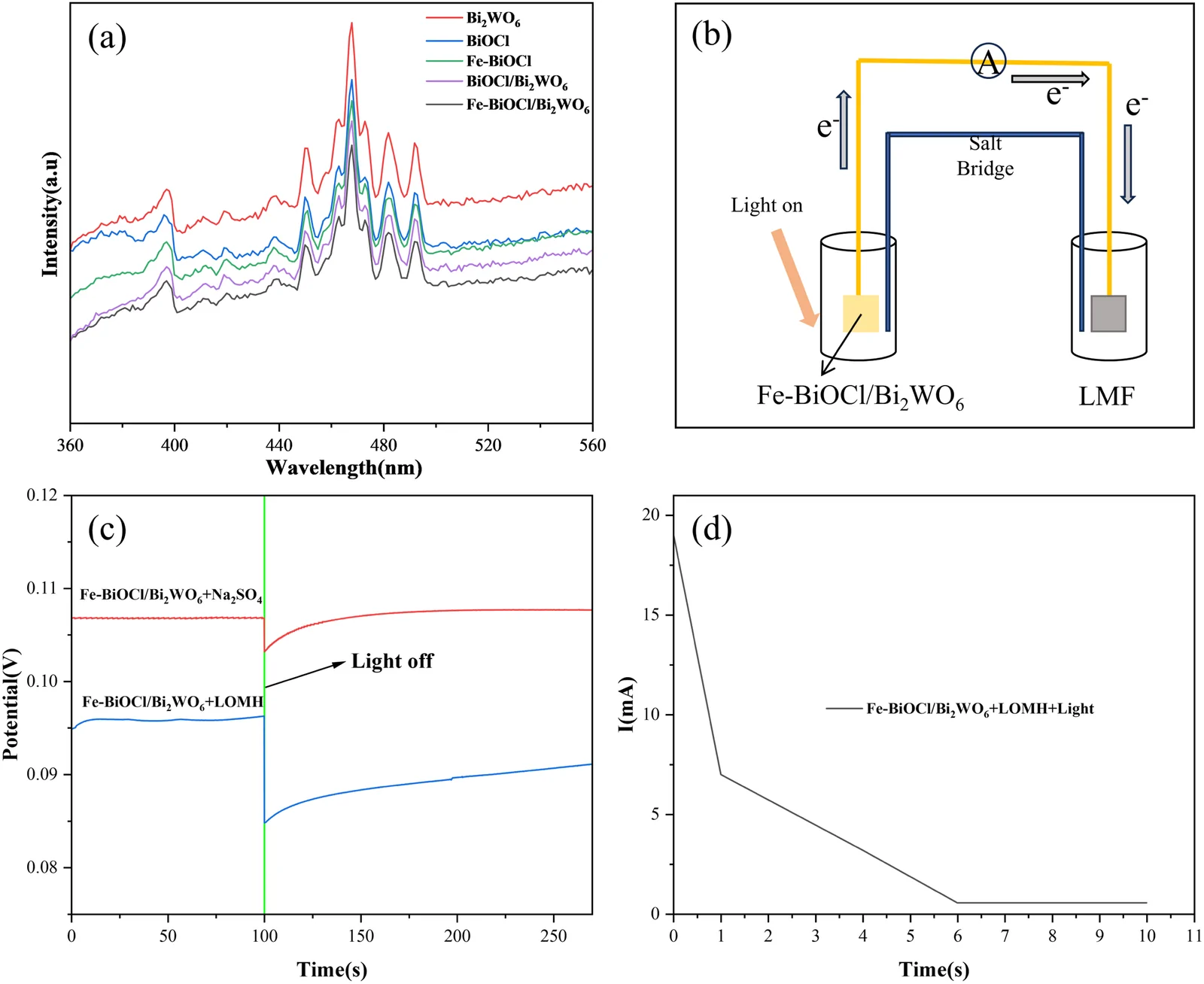
(a)Fluorescence spectra of BiOCl, Fe-BiOCl, Bi_2_WO_6_, BiOCl/Bi_2_WO_6_ and Fe-BiOCl/Bi_2_WO_6_; (b) schematic diagram of the electric oxidation system; (c) open circuit potential; (d) relative current in photocatalytic system.

The electronic band structures of the various photocatalysts were further investigated using Mott–Schottky measurements. As shown in Fig. S5, the consistent positive slopes in the Mott–Schottky plots unequivocally verify that all synthesized samples exhibit n-type behavior.^56^ The flat band potentials of Fe-BiOCl, BiOCl, Bi_2_WO_6_, and Fe-BiOCl/Bi_2_WO_6_ were measured to be sequentially −0.50 eV, −0.42 eV, −0.13 eV and −0.36 eV (*vs.* reference electrode). In n-type semiconductors, the Fermi level lies close to the conduction band, resulting in the conduction band edge being slightly more negative (by ∼0.2 eV) than the flat-band potential.^57^ Based on this relation, the calculated ECB values for BiOCl, Fe-BiOCl, Bi_2_WO_6_, and Fe-BiOCl/Bi_2_WO_6_ are sequentially −0.70 eV, −0.62 eV, −0.33 eV, and −0.56 eV. According to the relationship *E*_VB_ = *E*_CB_ + *E*_g_, in combination with the *E*_g_ values obtained from Tauc plots, the *E*_VB_ of Fe-BiOCl and Bi_2_WO_6_ are computed to be sequentially 1.43 eV and 2.29 eV.

While charge separation is evident, the precise pathway of charge carrier transfer remains unclear during photocatalytic process, an open-circuit potential (OCP) of FTO electrode coated with Fe-BiOCl/Bi_2_WO_6_ was measured without applying external bias. As shown in Fig. 4c, the xenon lamp was turned off during the initial 0–100 s period. Upon illumination at 100 s, the OCP abruptly decreased and subsequently stabilized. This behavior can be attributed to the rapid consumption of photogenerated electrons through reaction with LMF, as electrons are transferred from the catalyst to the pollutant molecules, they become depleted, leading to a reduction in the oxidation potential. The subsequent stabilization reflects a dynamic equilibrium between continuous charge carrier recombination and interfacial transfer.^58^

Furthermore, to corroborate the occurrence of charge transfer, a specially designed electrochemical bridge system incorporating a salt bridge was constructed (Fig. 4b). The current–time curve of this photocatalytic bridge system is presented in Fig. 4d. In the Fe-BiOCl/Bi_2_WO_6_ + LMF system, the continuous migration of electrons from catalyst to pollutant molecules resulted in a gradual decrease in current over time, consistent with electron consumption during the reaction.

### 3.2 Photocatalytic degradation of LMF

#### 3.2.1. Impact trial

The solution pH is a critical factor influencing catalytic performance. Hence, photocatalytic trials were performed to explore the impact of original pH on the LMF degradation using Fe-BiOCl/Bi_2_WO_6_. The experiments were performed under the following parameters: LMF (mg L^−1^), photocatalyst (0.2 g L^−1^), xenon lamp power of 500 W, and visible-light irradiation for 6 h. The pH was regulated by the addition of 0.1 M NaOH and HCl solutions prior to the addition of photocatalyst. It is displayed in Fig. 5a, the removal efficiency of LMF rose from 72% to 95% when the original pH grow from 3 to 7. Nevertheless, a subsequent rise in pH to 11 led to a decline in degradation efficiency to 75%. Under acidic conditions, the excessive H^+^ ions can scavenge ˙OH, thereby inhibiting the production of ROS and reducing LMF removal.^59^ As a fluoroquinolone antibiotic, LMF has dissociation constants (p*K*_a_) of 6.0 and 8.0. At pH < 6, LMF primarily present in the protonated (cationic) state, while at pH > 8, it is mainly in the anionic form ref. 60. Under highly alkaline conditions, like-charge repulsion between the LMF molecules and photocatalyst surface may prevent effective adsorption of LMF onto active sites, thus impairing degradation. Moreover, compared to alkaline conditions, a neutral pH is more desirable from the perspective of practical wastewater treatment and discharge.^61^ Based on these findings, a neutral pH of 7 was selected for all subsequent experiments.

**Fig. 5:**
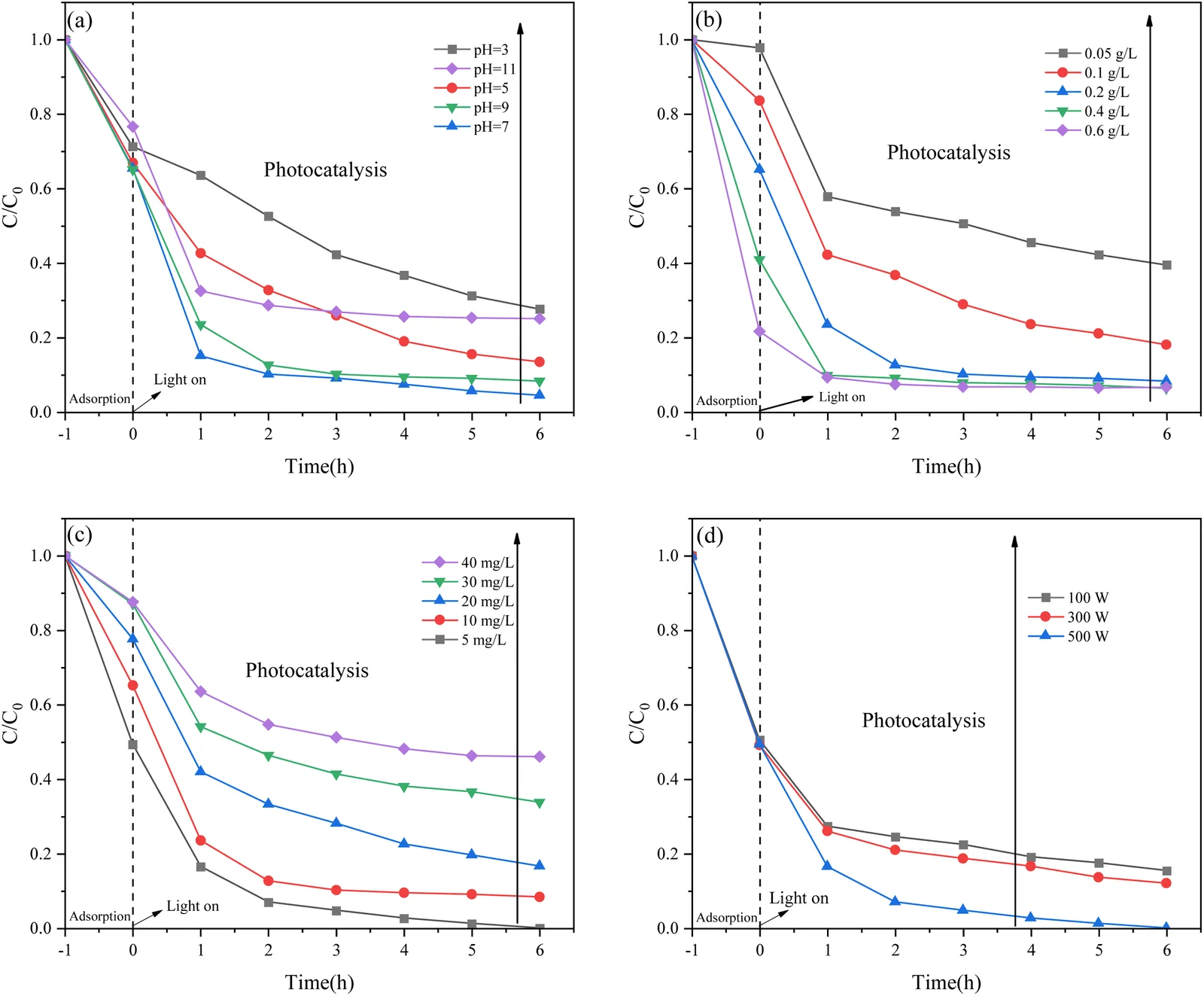
The effect of pH (a), catalysis dose (b), pollution content (c) and visible-light power (d) on LMF degradation trough Fe-BiOCl/Bi_2_WO_6_.

To elucidate the correlation between catalyst concentration and photocatalytic activity, trials were conducted by varying the amount of Fe-BiOCl/Bi_2_WO_6_ from 0.05 to 0.6 g L^−1^, building upon the previously optimized pH condition. As display in Fig. 5b, the degradation efficiency of LMF increased progressively with higher catalyst loadings. The observed activity boost is driven by an abundance of surface active sites and elevated yields of ROS, which promote the adsorption and decomposition of LMF.^61^ However, when an increase in photocatalyst dosage from 0.2 to 0.6 g L^−1^, although the adsorption in the dark phase improved noticeably, no significant further increase in photocatalytic removal was observed. This phenomenon is likely due to agglomeration and increased turbidity at higher catalyst concentrations, which reduce light penetration and hinder effective visible-light absorption.^62^ Therefore, considering both efficiency and practical applicability, we identified 0.2 g L^−1^ as the optimum catalyst concentration and adopted this value for all further experiments.

In addition to the initial pH and catalyst dosage, the influence of the initial concentration of LMF was also investigated. Building upon the previous catalyst dosage experiments, photocatalytic degradation was evaluated at five different LMF initial molality: 5, 10, 20, 30, and 40 mg L^−1^. As depicted in Fig. 5c, the degradation efficiency was inversely proportional to the initial concentration of LMF. Specifically, a removal rate of 100% was achieved within 6 hours under visible light irradiation at 5 mg L^−1^ original concentration of LMF. In contrast, the degradation efficiency reduced to 54% along with the starting molality was increased to 40 mg L^−1^. This decline may be ascribed to the limited mass transport and reduced accessibility of reactive sites to pollutant molecules at higher concentrations, which restrains the interaction between LMF and reactive species.^63^ Furthermore, intermediate products generated during the degradation of high concentrations of pollutants may accumulate on the catalyst surface, leading to active site saturation and thereby impeding the overall photocatalytic process.

Building upon the optimized parameters including solution pH, catalyst dosage, and initial concentration, the influence of light irradiation intensity on the photocatalytic system was further investigated. As illustrated in Fig. 5d, when the light intensity was reduced to 100 W and 300 W, the degradation efficiency of LMF decreased by 15% and 12%, respectively. Our findings pinpoint 500 W as the optimal light intensity to harness the photocatalytic potential of Fe-BiOCl/Bi_2_WO_6_ for LMF degradation. Based on the systematic investigation of these influencing factors, the theoretically optimal conditions for the degradation of LMF with Fe-BiOCl/Bi_2_WO_6_ are determined as follows: initial pH of 7.0, 5 mg per L LMF, 0.2 g per L catalyst, and light intensity of 500 W.

#### 3.2.2. Degradation kinetics

Fig. S6 presents the removal efficiencies of LMF by various catalysts under optimized degradation conditions. Based on the results summarized in Fig. 5, under the optimal experimental parameters, the removal rates of LMF reached 89.9%, 91%, 81.5%, 96%, and 100% for BiOCl, Fe-BiOCl, Bi_2_WO_6_, BiOCl/Bi_2_WO_6_, and Fe-BiOCl/Bi_2_WO_6_, respectively. The above results align with the fluorescence spectroscopy data discussed in Section 3.1.2. All other photocatalysts exhibited lower degradation efficiencies compared to Fe-BiOCl/Bi_2_WO_6_, indicating that the heterojunction generated from Fe-BiOCl and Bi_2_WO_6_ significantly enhances the photocatalytic activity.

At adsorption–desorption equilibrium, the dark adsorption removal rate of Fe-BiOCl/Bi_2_WO_6_ was 51%, which is lower than that of BiOCl/Bi_2_WO_6_ (64%). However, after 2 hours of visible light irradiation, Fe-BiOCl/Bi_2_WO_6_ demonstrated a higher overall removal efficiency for LMF. This suggests that Fe doping synergistically facilitates charge dissociation and suppresses bulk recombination. Upon light irradiation, a greater quantity of ROS were generated, thereby improving the removal efficiency of LMF.

### 3.3 Theoretical calculation

#### 3.3.1. Model construction and optimization

Fig. S7(a)–(c) display the optimized bulk structural models of BiOCl, Bi_2_WO_6_, and Fe-BiOCl, respectively. The lattice parameters of BiOCl are *α* = *β* = *γ* = 90°, *a* = *b* = 3.87 Å, *c* = 8.09 Å. Those of Bi_2_WO_6_ are *α* = *β* = *γ* = 90°, *a* = *c* = 5.53 Å, *b* = 16.70 Å. For Fe-BiOCl, the lattice parameters are *α* = *β* = *γ* = 90°, *a* = 11.59 Å, *b* = 7.73 Å, *c* = 18.72 Å. The Fe-BiOCl model was constructed from a 2 × 3 × 2 supercell of pristine BiOCl, in which a central Bi atom was substituted by an iron atom. The resulting structure contains 24 chlorine atoms, 32 oxygen atoms, and one iron atom, corresponding to an Fe doping concentration of 4.16%, which is consistent with the experimental value of approximately 5%. Based on TEM observations, the dominant exposed facets, namely BiOCl (111) and Bi_2_WO_6_ (112), were selected for surface modeling. The heterojunction interface was constructed by combining these two facets. A possible atomic configuration of the Fe-BiOCl/Bi_2_WO_6_ heterojunction is illustrated in Fig. 6. The composite model was built in two steps: first, Fe doping was introduced into the BiOCl lattice to form Fe-BiOCl, then, the Fe-BiOCl and Bi_2_WO_6_ surfaces were integrated to form the final heterostructure.

**Fig. 6:**
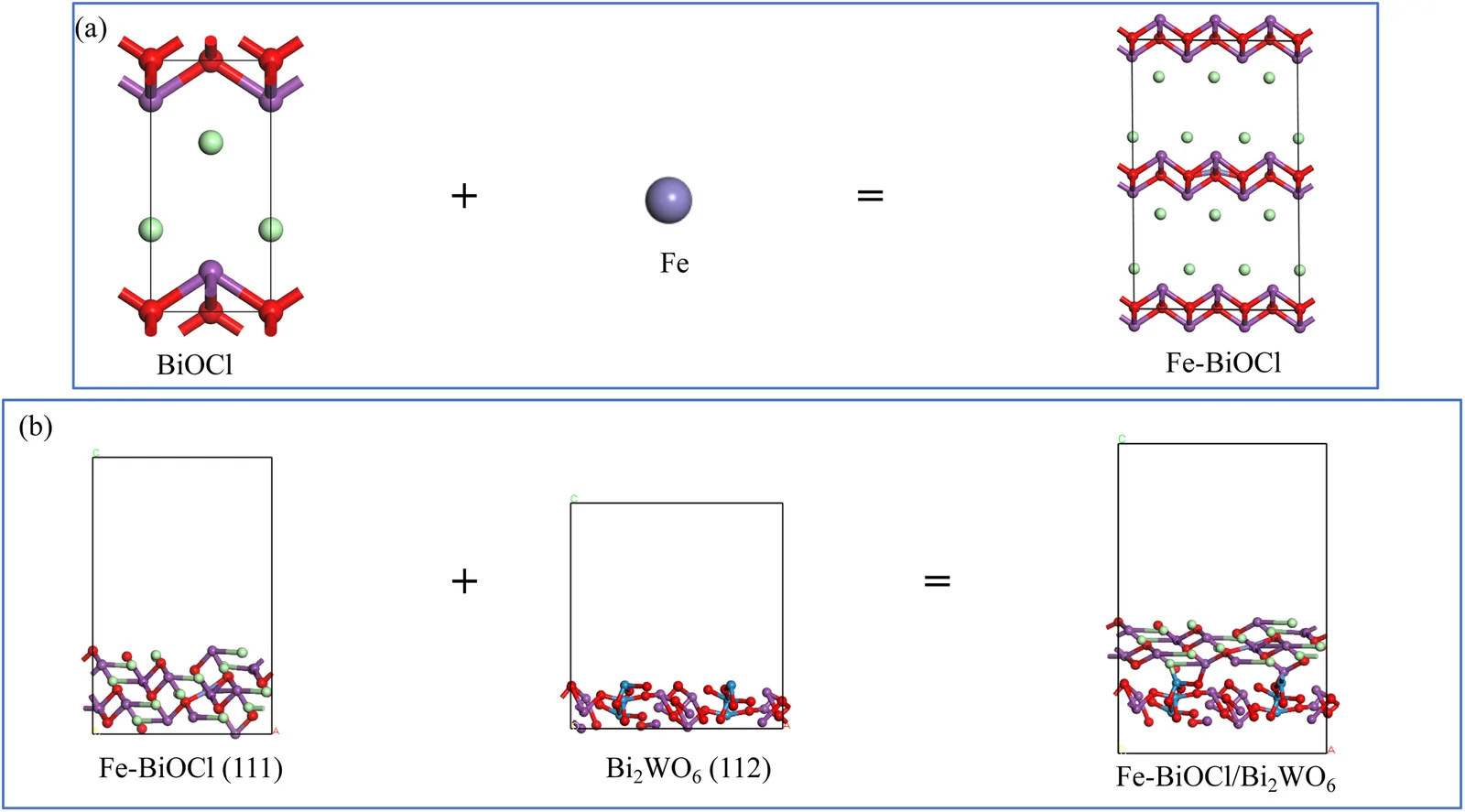
Recombination of heterojunction (a) Fe-BiOCl; (b) Fe-BiOCl/Bi_2_WO_6_.

#### 3.3.2. Electronic band structure

To gain further insight into the aforementioned processes, particularly the modifications to the electronic structure stemming from Fe incorporation, density functional theory (DFT) calculations were performed for Fe-BiOCl, BiOCl, and Bi_2_WO_6_. The calculated electronic band structures are presented in Fig. 7. The band gaps (*E*_g_) of Fe-BiOCl, BiOCl, and Bi_2_WO_6_ were determined to be 1.226 eV, 2.656 eV, and 2.139 eV, respectively. It should be noted that owing to the inherent limitations of the generalized gradient approximation, the absolute bandgap values are underestimated compared to experimental measurements. Nevertheless, the computational approach remains valid for analyzing trends in electronic properties and the effects of Fe doping on band structure evolution. The results clearly indicate a significant narrowing of the band gap after Fe incorporation, suggesting that Fe^3+^ influences the recombination processes of electrons and holes. Specifically, Fe^3+^ can serve as a trapping center for both charge carriers: it could be reduced to Fe^2+^ by capturing electrons and subsequently reoxidized to Fe^3+^ by molecular oxygen, thereby facilitating electron transfer and suppressing charge recombination. Thus, an appropriate concentration of Fe^3+^ doping promotes electron–hole pair dissociation, leading to a significant boost in photocatalytic activity.

**Fig. 7:**
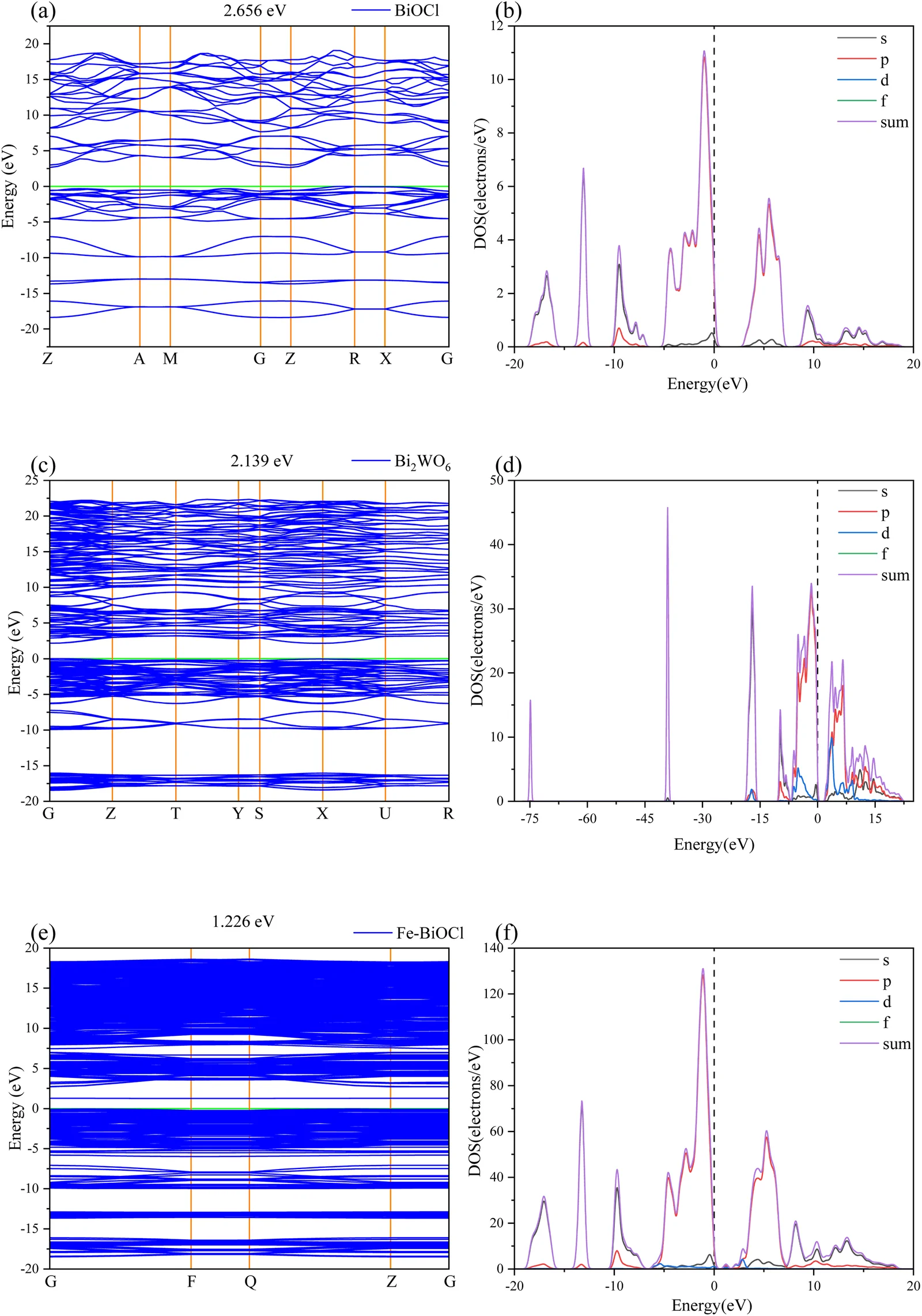
Band diagram of (a) BiOCl, (b) Bi_2_WO_6_ and (c) Fe-BiOCl; state density diagram of (d) BiOCl, (e) Bi_2_WO_6_ and (f) Fe-BiOCl.

Furthermore, the density of states-including total density of the states (TDOS) and projected density of states (PDOS)-was investigated to elucidate the contributions of different electronic orbitals to valence band (VB) and conduction band (CB). For pristine BiOCl, VB is primarily consists of Bi 6p, Cl 2p and O 2p orbitals, whereas CB consists mainly of O 2p and Bi 6p orbitals.^64^ After Fe doping, additional states attributed to Fe 3d orbitals appear near the Fermi level. According to d-band theory, these states enhance the catalytic performance of the photocatalyst.^53^ For Bi_2_WO_6_, the TDOS and PDOS analyses reveal that the VB is dominated by Bi 6s, O 2p, and W 5d orbitals, whereas the CB comprises contributions from Bi 6s, W 5s, O 2p, W 5p, and Bi 6p orbitals.^65^

Specifically, DRS provides the visible-light absorption range and band-gap values. Mott–Schottky determines the flat-band potentials and band-edge positions, establishing the thermodynamic driving force for interfacial charge transfer; and DFT reveals the electronic-structure origin, including the Fe 3d states introduced by doping and the interfacial built-in electric field that directs electron and hole migration. Together, these results support a consistent charge-transfer mechanism in which Fe doping and heterojunction formation synergistically promote spatial charge separation and suppress recombination.

#### 3.3.3. Adsorption energy

The adsorption/desorption behavior of reactants is a critical determinant of catalytic efficiency.^66^ To quantify this interaction in the context of LMF photodegradation, DFT calculations were performed to model the adsorption of LMF on the Fe-BiOCl/Bi_2_WO_6_ surface. The optimized adsorption configurations are depicted in Fig. 8. The calculated adsorption energies reveal a significant configuration-dependent interaction: an energy of −0.49 eV was obtained when LMF adsorbs with its reactive moiety oriented toward the catalyst surface (“face-up”), compared to −0.90 eV for the opposite orientation (“face-down”). The more negative the adsorption energy, the stronger the adsorption. The more negative adsorption energy for the face-down configuration indicates a thermodynamically more favorable and potentially faster adsorption–desorption equilibrium, which is conducive to the subsequent photocatalytic oxidation of LMF. In the down configuration, the adsorbate interacts more directly with the exposed active sites, allowing stronger orbital overlap and more effective charge transfer between the adsorbate and the catalyst surface. The face-up configuration may suffer from stronger steric hindrance or less favorable orbital orientation, weakening the adsorption strength. The adsorption energy was determined *via* the following expression, which is defined as:^67^

1 *E*_ads_ = *E*_surface+pollutant_ − *E*_surface_ − *E*_pollutant_

*E*_ads_ means the adsorption energy between pollutants and catalyst surfaces, *E*_surface+pollutant_ means the energy of the catalyst and pollutants, *E*_surface_ means the surface energy of the catalyst, *E*_pollutant_ means the energy of pollution.

**Fig. 8:**
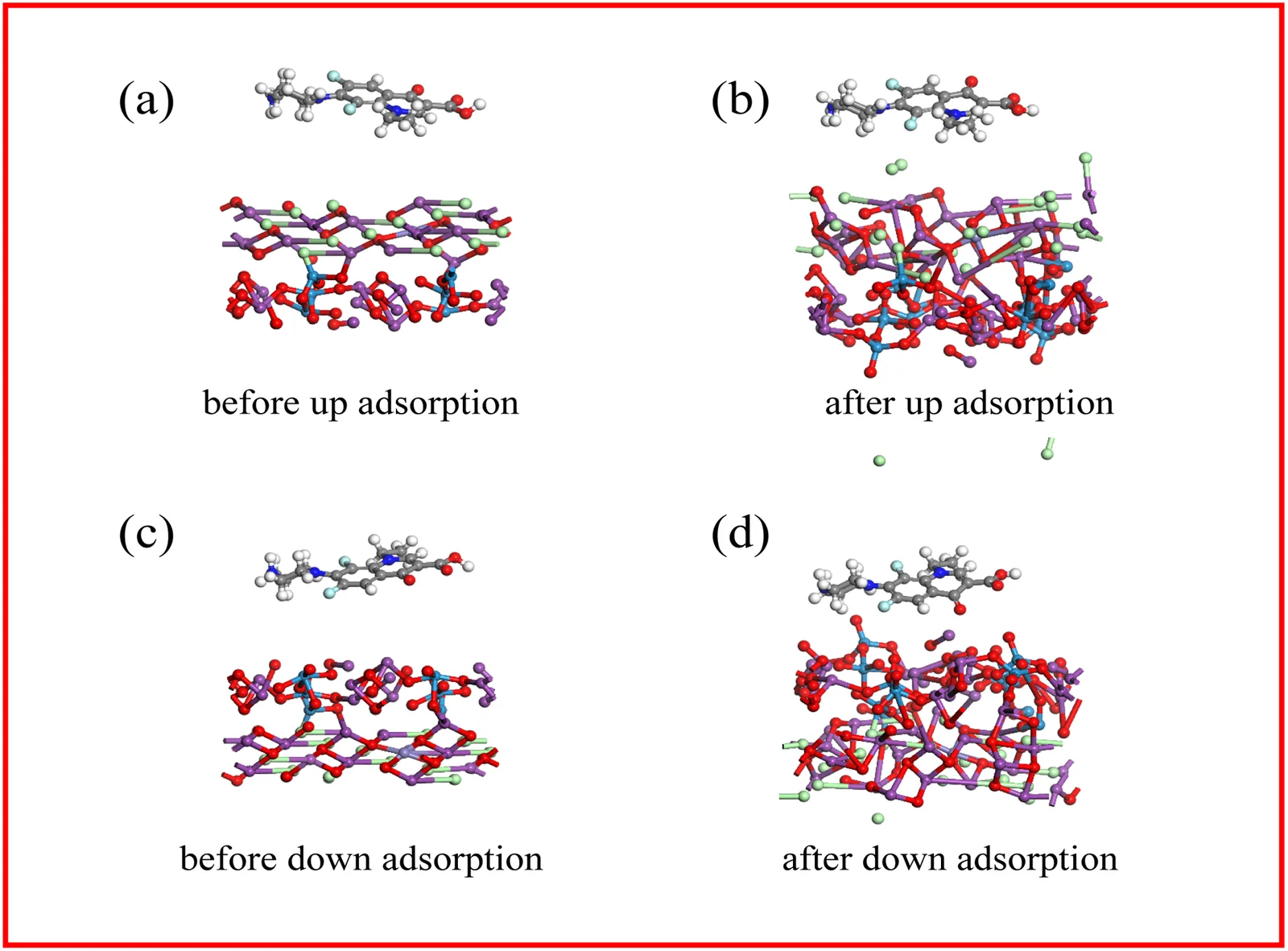
The adsorption model of LMF on the Fe-BiOCl/Bi_2_WO_6_ surface.

### 3.4 Mechanism analysis

#### 3.4.1. Quenching experiments

Radical quenching experiments are a fundamental qualitative approach for investigating photocatalytic mechanisms. In this study, sodium oxalate (Na_2_C_2_O_4_), *p*-benzoquinone (BQ), and *tert*-butanol (*t*-BuOH) were employed as specific quenchers for h^+^, ˙O_2_^−^, and ˙OH, respectively. The trials were conducted under the optimal conditions to identify the reactive species involved during the photocatalytic process. As displayed in Fig. 9a, the degradation efficiency of LMF by Fe-BiOCl/Bi_2_WO_6_ decreased by 18.4%, 8.5%, and 5.5% with the addition of 10 mM, 5 mM, and 1 mM sodium oxalate, respectively, implying that h^+^ possess a irreplaceable function overall the photocatalytic process. Similarly, it can be seen in Fig. 9b, the degradation was partially suppressed upon the introduction of *t*-BuOH. The removal rate of LMF declined by 12.6% when 10 mM *t*-BuOH was introduced, suggesting that ˙OH supplemented the removal effect of LMF. Furthermore, as presented in Fig. 9c, the removal efficiency of LMF gradually declined with increasing concentration of BQ. The inhibition rates reached 16.5%, 12.0%, and 4.2% at BQ molarity of 10 mM, 5 mM, and 1 mM, respectively, confirming that ˙O_2_^−^ is another primary ROS during the photocatalytic degradation process of LMF. In conclusion, ˙O_2_^−^, ˙OH, and h^+^ collectively generated by the Fe-BiOCl/Bi_2_WO_6_ catalyst contribute to the degradation of LMF, breaking it down into smaller intermediates and ultimately mineralizing them into CO_2_ and H_2_O.

**Fig. 9:**
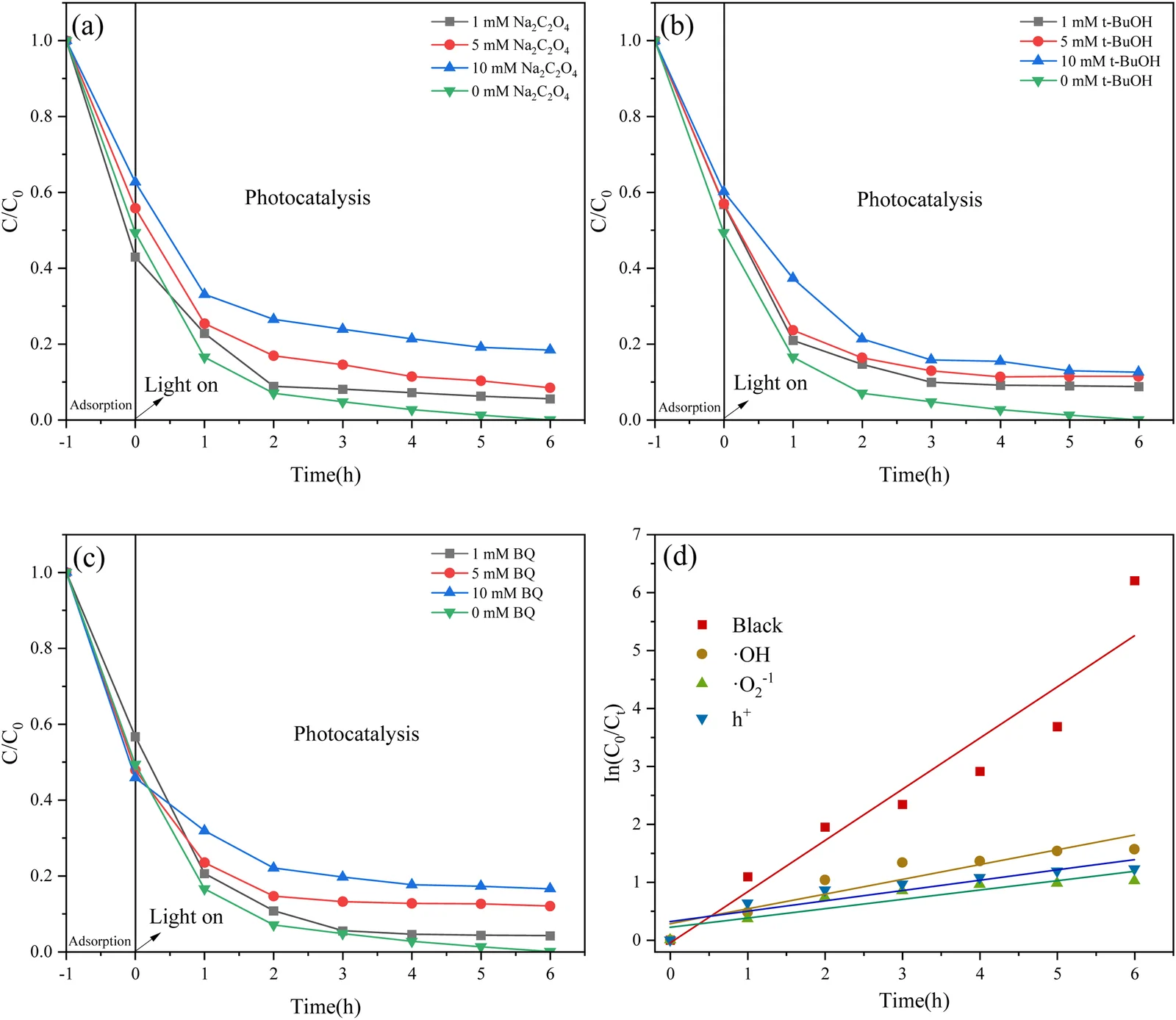
Effects of different concentrations of (a) sodium oxalate, (b) *tert*-butanol, (c) *p*-benzoquinone on the degradation of LMF by Fe-BiOCl/Bi_2_WO_6_; (d) free radical contribution.

To quantify the proportions of various ROS to the photocatalytic degradation reaction of LMF, the apparent pseudo-first-order kinetics were fitted to determine the degradation rates following the introduction of various radical scavengers. The results are presented in Fig. 9d. In the absence of any quencher, the degradation rate constant of the blank group was 0.6933 h^−1^. After adding the quenchers Na_2_C_2_O_4_, *t*-BuOH, and BQ, the rate constants decreased sequentially to 0.1783 h^−1^, 0.255 h^−1^, and 0.1612 h^−1^. The contribution ratio of each active species was calculated using the following equation:^68^
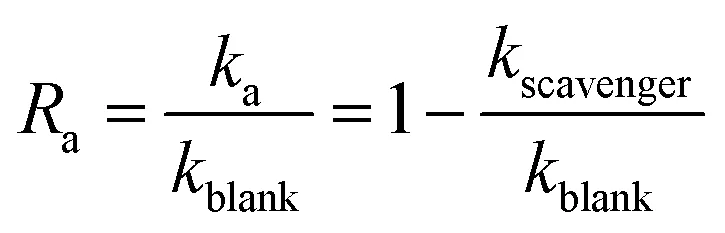
where ‘a’ denotes the type of active species, *k*_scavenger_ represents the degradation rate constant of LMF upon addition of quenchers, *k*_blank_ is the degradation rate constant without quencher. The results indicate that ˙OH, ˙O_2_^−^, and h^+^ contributed approximately 63.2%, 76.75% and 74.3%, respectively, to the overall degradation.

To further verify the types of radicals generated in the photocatalytic system, spin-trapping EPR spectroscopy with DMPO was utilized to directly detect the presence of ˙O_2_^−^.^69^ As shown in Fig. S8, after 10 minutes of light irradiation, a characteristic signal consisting of ‘four strong and two weak’ peaks was detected, demonstrating the generation of substantial superoxide radicals during the photocatalytic process. This data is in agreement with the findings from the radical quenching experiments, further supporting that superoxide radicals as the primary reactive species during the photocatalytic process.

Specifically, the CB of Bi_2_WO_6_ lies more negative than the VB of Fe-BiOCl, allowing electrons from the CB of Bi_2_WO_6_ to recombine with holes from the VB of Fe-BiOCl at the interface, while the CB electrons of Fe-BiOCl and VB holes of Bi_2_WO_6_ are preserved. This is consistent with the detected ˙O_2_^−^ (76.75%), h^+^ (74.28%), and ˙OH (63.22%), since the preserved CB of Fe-BiOCl is negative enough to reduce O_2_ to ˙O_2_^−^ and the preserved VB of Bi_2_WO_6_ is positive enough to oxidize water/OH^−^ to ˙OH. A conventional type-II pathway would contradict these observations. The graphical abstract clearly shows the Z-scheme interfacial recombination pathway and the preserved reductive and oxidative sites, with the corresponding redox potentials indicated.

#### 3.4.2. Analysis of degradation products and pathway simulation

The molecular structure of LMF was constructed and computationally analyzed using the Dmol^3^ module in Materials Studio. The electron density distribution of frontier molecular orbitals (FMOs), including the lowest unoccupied molecular orbital (LUMO) and the highest occupied molecular orbital (HOMO), supplies critical understanding of various reaction types. In general, the LUMO functions as the primary electron acceptor, whereas the HOMO readily donates electrons. A higher HOMO energy facilitates electron loss, whereas a lower HOMO energy favors electron acceptance.^70^

As presented in Fig. 10a, the HOMO electron density of LMF is primarily localized on the piperazine ring and fluorine atom, suggesting that these regions are vulnerable to electrophilic oxidation by radicals such as h^+^ and ˙OH. In contrast, the LUMO distribution is concentrated around the quinoline ring and its adjacent functional groups, suggesting vulnerability to nucleophilic radicals like ˙O_2_^−^.^71,72^

**Fig. 10:**
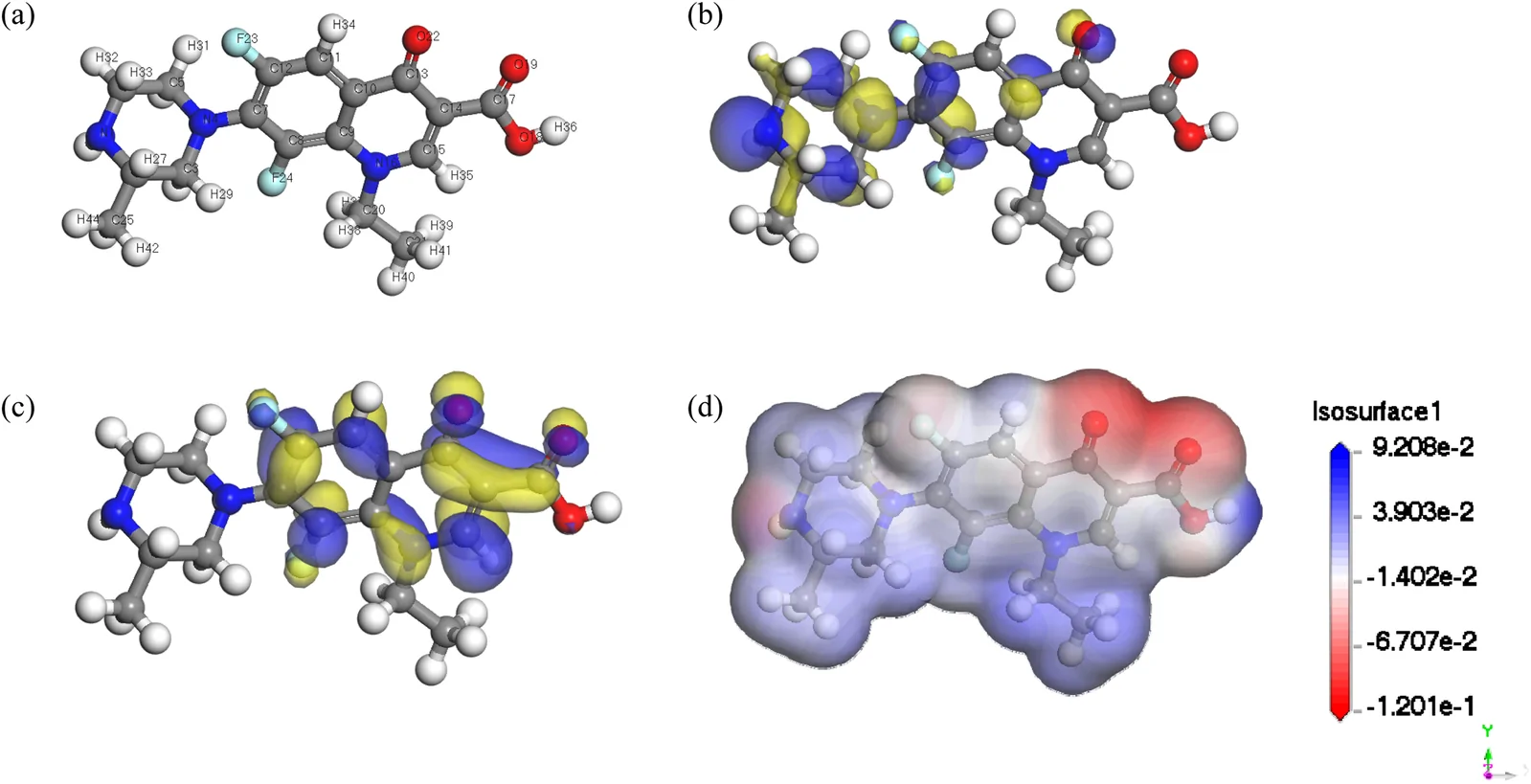
(a) Numbered chart of LMF; (b) HOMO orbital; (c) LUMO orbital; and (d) electrostatic potential of LMF.

Electrostatic potential (ESP) analysis further elucidates the reactive sites for electrophilic and nucleophilic attacks. As depicted in Fig. 10d, regions with partial positive charge are indicated in blue, while partially negative regions are shown in red. The O22 and O19 atoms in LMF exhibit negative ESP (red), rendering them prone to electrophilic assault. Conversely, atoms such as C2, C20, C21, C3, C25, C6, and their bonded hydrogen atoms display positive ESP (blue), indicating susceptibility to nucleophilic attack.

To gain deeper insights into the detailed decomposition process of LMF using Fe-BiOCl/Bi_2_WO_6_ photocatalysis, the key intermediates were characterized through LC-MS/MS. Combined with DFT calculations, the decomposition pathways of LMF were advanced. It was illustrated in Fig. 11, in part 1, the LMF molecule undergoes defluorination and hydroxylation, yielding intermediate P1 (*m*/*z* = 288). Under continued radical attack, the N–C bond connecting the piperazine ring is broken, leading to the separation of the piperazine and quinoline rings. The quinoline ring then undergoes demethylation, dehydroxylation, and decarbonylation, forming P2 (*m*/*z* = 149). In the pathway denoted as part 2, intermediate P3 (*m*/*z* = 382) is generated *via* ring-opening of the piperazine moiety in LMF.^73^ P3 is subsequently transformed through oxidation, hydroxylation, defluorination, and carbon-chain cleavage into P4 (*m*/*z* = 338). Alternatively, P8 (*m*/*z* = 338), which shares the same mass-to-charge ratio, can be directly formed from LMF through ring cleavage, hydroxyl substitution, and C–C bond scission.^71^ Decarboxylation of P4 promotes the generation of P5 (*m*/*z* = 294), which is further degraded to simpler molecular species P6 (*m*/*z* = 136) and P7 (*m*/*z* = 60). Regarding part 3, cleavage of the N–C bond between the piperazine and quinoline rings readily occurs, resulting in P9 (*m*/*z* = 255).^74^ Under persistent ROS attack, side-chain C–C bonds connected to the quinoline structure are broken, producing P10 (*m*/*z* = 178) and P11 (*m*/*z* = 150). With the photoreaction proceeds, the large molecular intermediates are progressively mineralized into small molecules and ultimately oxidized by h^+^, ˙OH, and ˙O_2_^−^ to CO_2_, H_2_O, F^−^, and other inorganic end products.

**Fig. 11:**
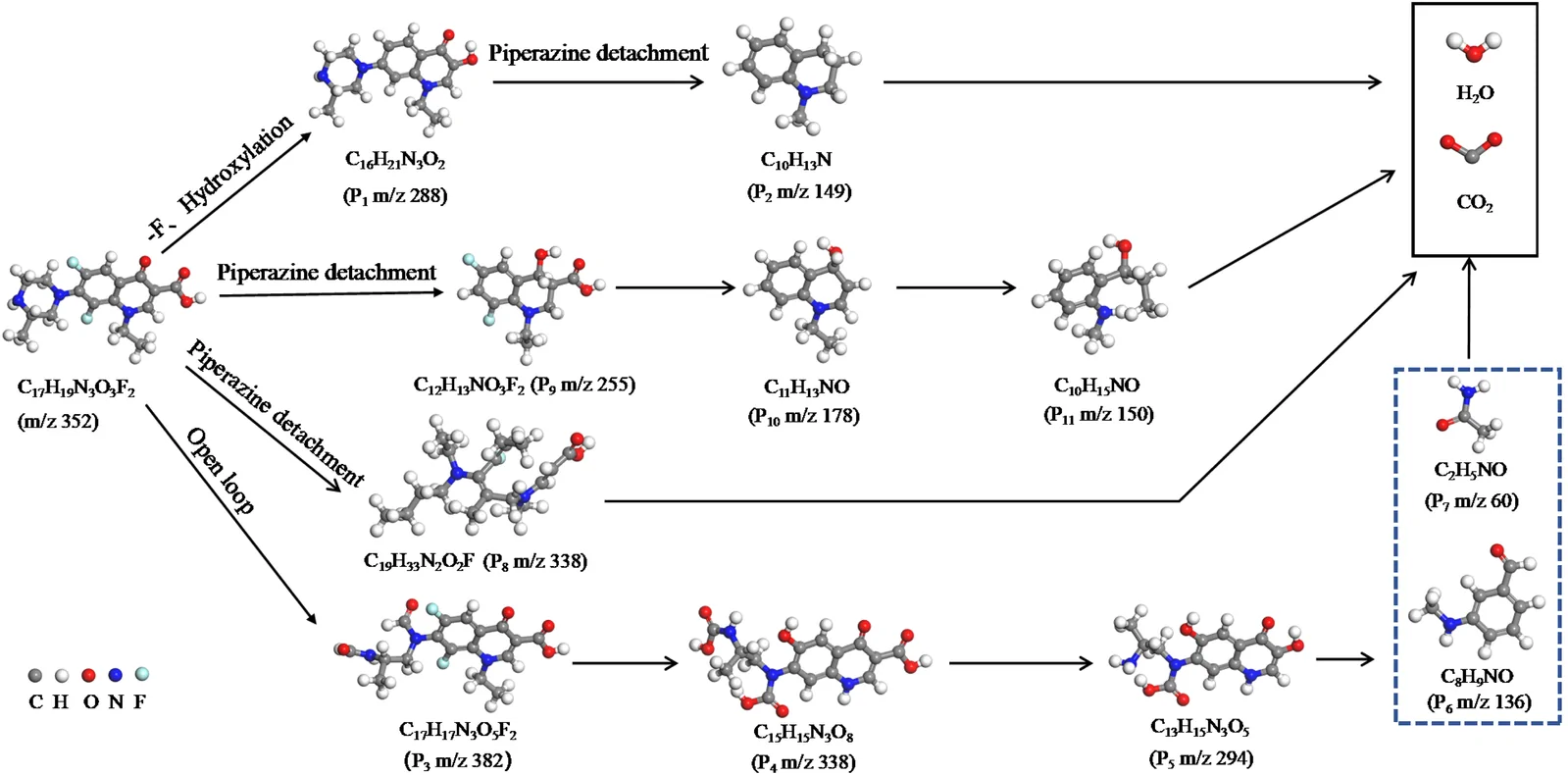
Pathway of Fe-BiOCl/Bi_2_WO_6_ photocatalytic degradation of LMF.

We used T.E.S.T software to predict the toxicity of LMF and its photocatalytic degradation intermediates, assessing acute toxicity (LC_50_, 96 h), developmental toxicity, bioaccumulation, and mutagenicity. LMF showed high acute toxicity to blackhead soft mouth bream (LC_50_ = 0.62 mg L^−1^). After degradation, all intermediates had lower acute toxicity than LMF as Fig. S10 shown, P7 was classified as non-toxic (LC_50_ = 1092.96 mg L^−1^). Developmental toxicity of all intermediates was reduced, with P6 showing no toxicity (0.45). While P1, P2, P9, and P10 had high bioaccumulation factors, P5, P6, and P8 showed low or no bioaccumulation. Mutagenicity decreased for all intermediates except P1, with LMF at 0.76 and others lower. Overall, photocatalysis effectively reduced the toxicity of LMF.

### 3.5 Evaluation of photocatalyst practicality

The critical attributes of practical photocatalysts are their long-term stability and reusability, which govern commercial viability. The photostability and reusability of the catalyst were evaluated through repeatedly utilizing the same batch of material for the photodegradation of LMF. Additionally, since the leaching of Fe ions during the photocatalytic process may negatively impact recyclability, the stability of photocatalyst was appraised through inductively coupled plasma mass spectrometry (ICP-MS).

After 6 hours of photocatalytic degradation of LMF, the Fe-BiOCl/Bi_2_WO_6_ composite was recovered through repeated washing with ethanol and distilled water, followed by filtration, collection, and drying. This material was then reused in subsequent photocatalytic cycles. As displayed in Fig. S9, a marginal decrease in degradation efficiency was observed with each cycle; nevertheless, over five successive runs, the degradation efficiency persisted as high as 92.0%, demonstrating excellent reusability of Fe-BiOCl/Bi_2_WO_6_.

The contents of Bi and Fe ions in the solution after 6 hours of photocatalytic degradation of LMF were quantified *via* ICP-MS. As summarized in Table S3, only minimal leaching of Fe and Bi ions was detected, indicating that the catalyst maintained a highly stable structure throughout the photocatalytic process.

## 4 Conclusion

In response to the challenge posed by LMF removal, this work developed an Fe-doped BiOCl/Bi_2_WO_6_ heterojunction photocatalyst for efficient visible-light-driven degradation. The central innovation lies in the synergistic coupling of Fe doping and interfacial heterojunction engineering, which together extend visible-light absorption, promote interfacial charge transfer, and suppress charge recombination—an effect unattainable by either strategy alone. Comprehensive characterization and systematic optimization of the iron doping level and component ratio establish a clear structure–activity relationship, while comparative experiments confirm that the enhancement arises from this synergy rather than from a simple combination of the individual components. Mechanistic studies show that ˙O_2_^−^ and h^+^ dominate the degradation process, with ˙OH playing an assisting role, achieving 47.43% mineralization of LMF into CO_2_ and H_2_O. Crucially, by correlating first-principles calculations with experimental evidence, this work reveals the key interfacial charge transfer pathways and reaction intermediates, providing a mechanistic framework for how Fe doping and heterojunction engineering jointly govern charge separation and reactive species generation. This work thus not only provides an efficient photocatalyst for LMF removal but also establishes a general design principle for doped heterojunction systems with tailored charge-transfer behavior, offering new insights into the rational construction of visible-light-driven photocatalysts for refractory organic pollutant degradation. The study further encompassed a proposed degradation pathway for LMF, an assessment of the ecotoxicity of its predicted intermediates, and an investigation into the influence of common inorganic anions (simulating real water matrices) on the degradation efficiency.

## Author contributions

Xinhong Gan: writing – original draft, funding acquisition, methodology. Qingquan Zhang: methodology, data curation, formal analysis. Yuqing Chen: writing – review & editing, data curation, formal analysis. Junyang Du: methodology, data curation. Li Sun: formal analysis, investigation. Jianhua Yang: formal analysis, conceptualization, writing – review & editing.

## Conflicts of interest

The authors declare that they have no known competing financial interests or personal relationships that could have appeared to influence the work reported in this paper.

## Supplementary Material

RA-OLF-D6RA06407H-s001

## Data Availability

The data that underpin the findings of this work are fully included within the article and its supplementary information (SI) files, with no additional external datasets required. Supplementary information: characterization data and reusability of the catalyst are detailed in the supplementary document. See DOI: https://doi.org/10.1039/d6ra06407h.
