# Supplementary material for: Absolute lomefloxacin degradation over visible-light-driven heterojunction: experimental and theoretical insights
Source: RSC Adv. 2026 Oct 12;16(47):62388–405. doi: 10.1039/d6ra06407h (PMC13656221; doi:10.1039/d6ra06407h)
Supplement: Supplementary file 1 [file RA-016-D6RA06407H-s001.pdf]

Supplementary Information (SI) for RSC Advances.
This journal is © The Royal Society of Chemistry

Absolute Lomefloxacin Degradation over Visible-Light-Driven Heterojunction: Experimental and Theoretical Insights

Xinhong Gan [a], Qingquan Zhang[b], Yuqing Chen[c], Junyang Du[a], Li Sun[a], Jianhua Yang[c*]

[a] State Environmental Protection Key Laboratory of Soil Environmental Management and Pollution Control, Nanjing Institute of Environmental Science, Ministry of Ecology and Environment (MEE) of China, Nanjing 210042 (China).

[b] Jiangsu Academy of Environmental Industry and Technology Corp, Nanjing, 210019, China.

[c] Anhui University of Technology, School of Energy and Environment, Maanshan 243002, Anhui, Peoples R China.

*Corresponding author:

Jianhua Yang, PHD

Anhui University of Technology, School of Energy and Environment, Maanshan 243002, Anhui, Peoples R China.

E-mail: yangjh@ahut.edu.cn

Text, Figure and Table captions

Text S1 Representing instrument information

Text S2 DFT calculation

Fig.S1. Effect of different composite ratios on the photocatalytic performance of Fe-BiOCl/Bi_2WO_6 (a); Influence of Fe Doping Content on the Photocatalytic Performance of Fe-BiOCl/Bi_2WO_6 (b)

Fig.S2. (a)X-ray diffraction patterns of Fe-BiOCl/Bi_2WO_6, Bi_2WO_6, Fe-BiOCl, BiOCl and BiOCl/Bi_2WO_6 and (b)Enlarged view of parts of Fe-BiOCl and BiOCl

Fig.S3. Fourier infrared spectra of Fe-BiOCl/Bi_2WO_6, Bi_2WO_6, Fe-BiOCl, BiOCl and BiOCl/Bi_2WO_6

Fig.S4. (a) N_2 adsorption-desorption isotherm diagram and (b) pore size distributions diagram of Fe-BiOCl/Bi_2WO_6, Bi_2WO_6, Fe-BiOCl, BiOCl and BiOCl/Bi_2WO_6

Table.S1. BET data for Fe-BiOCl/Bi_2WO_6、Fe-BiOCl、BiOCl、Bi_2WO_6 and BiOCl/Bi_2WO_6

Fig.S5. Mott-Schottky plots of (a)BiOCl, (b)Fe-BiOCl, (c)Bi_2WO_6 and (d)Fe-BiOCl/Bi_2WO_6

Fig.S6. Fe-BiOCl/Bi_2WO_6、Fe-BiOCl、BiOCl、Bi_2WO_6 and BiOCl/Bi_2WO_6 deoxidized LMF at 500 W visible light

Fig.S7. EPR spectra at dark and after 10 minutes under visible-light irradiation of LMF degradation by Fe-BiOCl/Bi_2WO_6

Table.S2 DFT-calculated theoretical adsorption energy values

Fig.S8. Pathway of Fe-BiOCl/Bi_2WO_6 photocatalytic degradation of LMF

Fig.S9. The effect of cyclic Fe-BiOCl/Bi_2WO_6 photocatalytic degradation of LMF

Fig.S10. Toxicity prediction of T.E.S.T. (a) Acute toxicity; (b) Developmental toxicity; (c) bioaccumulation factors; (d) Mutagenicity of LMF and degradation intermediates.

Fig.S11. Dark adsorption process of LMF on Fe-BiOCl/Bi_2WO_6 and (b) natural photolysis of LMF

Fig.S12. Photocatalytic kinetic fitting results of LMF by Bi_2WO_6, BiOCl, Fe-BiOCl, BiOCl/Bi_2WO_6, and Fe-BiOCl/Bi_2WO_6 at 500 W visible light

Table.S3. Leaching concentration of metal ions in solution after Fe-BiOCl/Bi_2WO_6 photocatalytic degradation

Table.S4. Mineralization rate of LMF by photocatalytic degradation of each catalyst

Fig.S13. Preparation process of composite catalyst

Text S1 Representing instrument information

The morphology and structure of the composite catalyst were analyzed using scanning electron microscopy (X-max 80 JSM7800F) and transmission electron microscopy (JEM-2100), and energy dispersive spectroscopy (EDS) was used to obtain the distribution maps of different elements in the material. The BET tester (ASAP 2460) was used with nitrogen as the adsorption and desorption medium, and the Barrett Joyner Halenda (BJH) theory was employed to analyze the specific surface area and pore size distribution curve of the photocatalyst. The crystal structure was analyzed using an XRD diffractometer (D8 ADVANCE) at 10-90°. Characterize the functional group characteristics of the catalyst using a Fourier transform infrared spectrometer (Nicolet 6700) with a wavelength range of 400-4000 cm-1. Characterize the various elements and their chemical states of Fe-BiOCl/Bi_2WO_6 using X-ray photoelectron spectroscopy (Thermo Scientific K-Alpha0 test). The light absorption characteristics of different samples were investigated by UV vis diffuse reflectance absorption spectroscopy, and the bandgap width was calculated. The wavelength range was measured from 200 to 800 cm. The photoluminescence spectrum of the photocatalyst was tested by a steady-state fluorescence spectrometer (FLS1000) under excitation light with a wavelength of 370 nm. On the CHI760E electrochemical workstation (Shanghai, China), the photocatalytic performance and carrier separation efficiency of the photocatalyst Fe-BiOCl/Bi_2WO_6 were evaluated using chronoamperometry, Mott Schottky experiment, electrochemical impedance spectroscopy, and open circuit potential measurement. High performance liquid chromatography quadrupole time-of-flight mass spectrometer (UPLC-QTOF-MS) (Agilent UPLC-1290 and Agilent QTOF-6550 combined) was used to detect intermediate products.

Text S2 DFT calculation

We performed the calculations within the generalized gradient approximation (GGA) framework for structural optimization and subsequent electronic structure, as parameterized by the

Perdew-Burke-Ernzerhof (PBE) functional. Vanderbilt-type ultrasoft pseudopotentials were adopted, with a plane-wave energy cutoff set to 500 eV. The geometric convergence criteria were defined as follows: maximum force ≤ 0.03 eV/Å, maximum energy change ≤ 1×10^{-5} eV/Å, maximum displacement ≤ 0.001 Å, and maximum stress ≤ 0.05 GPa. Electronic minimization was conducted using a density mixing scheme, and the self-consistent field (SCF) convergence threshold was set to 1×10^{-6} eV/atom. All atomic positions within the unit cell were fully relaxed without any constraints. For the structural relaxation, a 15×5×15 Monkhorst-Pack k-point grid was employed for sampling the Brillouin zone. Atomic charges and spin density distributions were analyzed using the grid-based Bader charge analysis method. The structural model was constructed with a 15 Å vacuum thickness along the c-axis to avoid interlayer interactions. The electronic band structure was interpreted via partial density of states (PDOS) analysis. All energies and atomic structures were fully optimized at each step of the calculation.

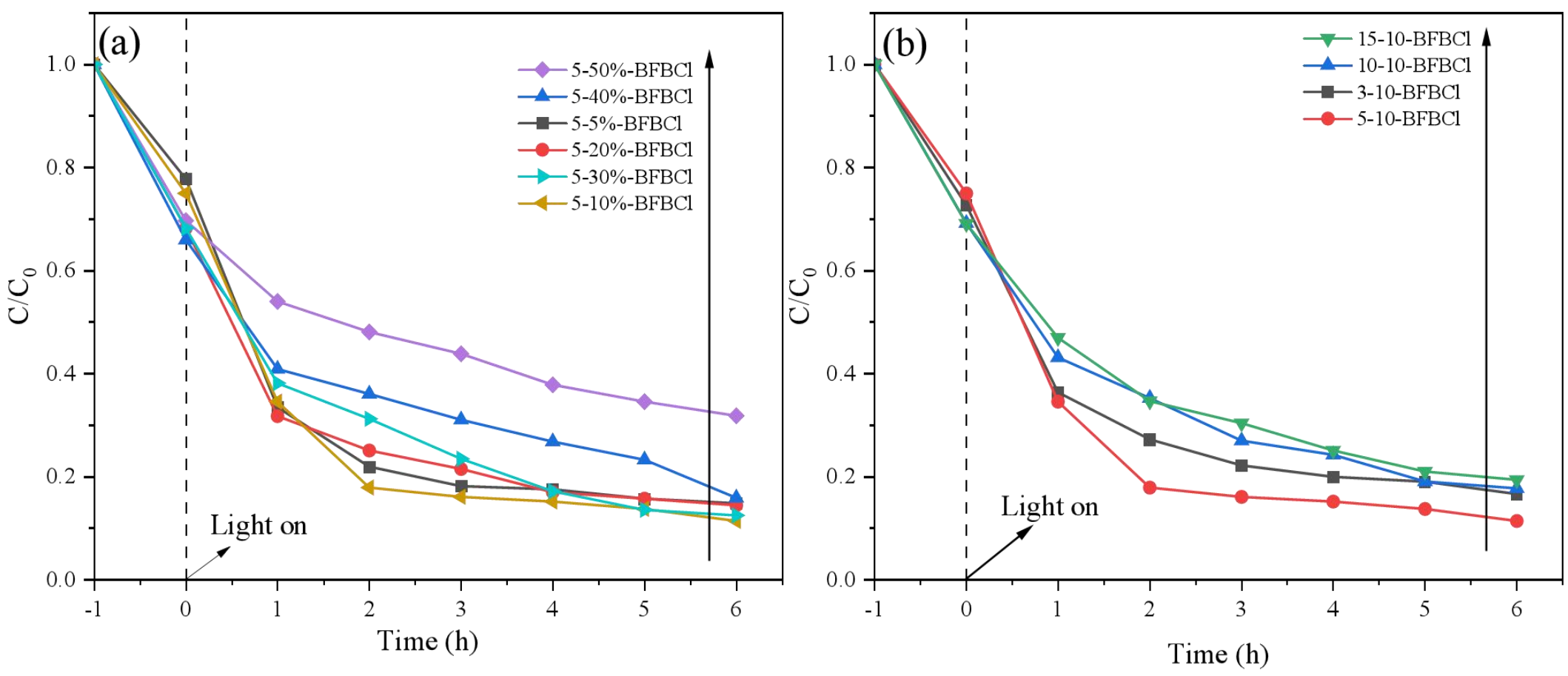

Fig.S1. Effect of different composite ratios on the photocatalytic performance of Fe-BiOCl/Bi_2WO_6 (a); Influence of Fe Doping Content on the Photocatalytic Performance of Fe-BiOCl/Bi_2WO_6 (b)

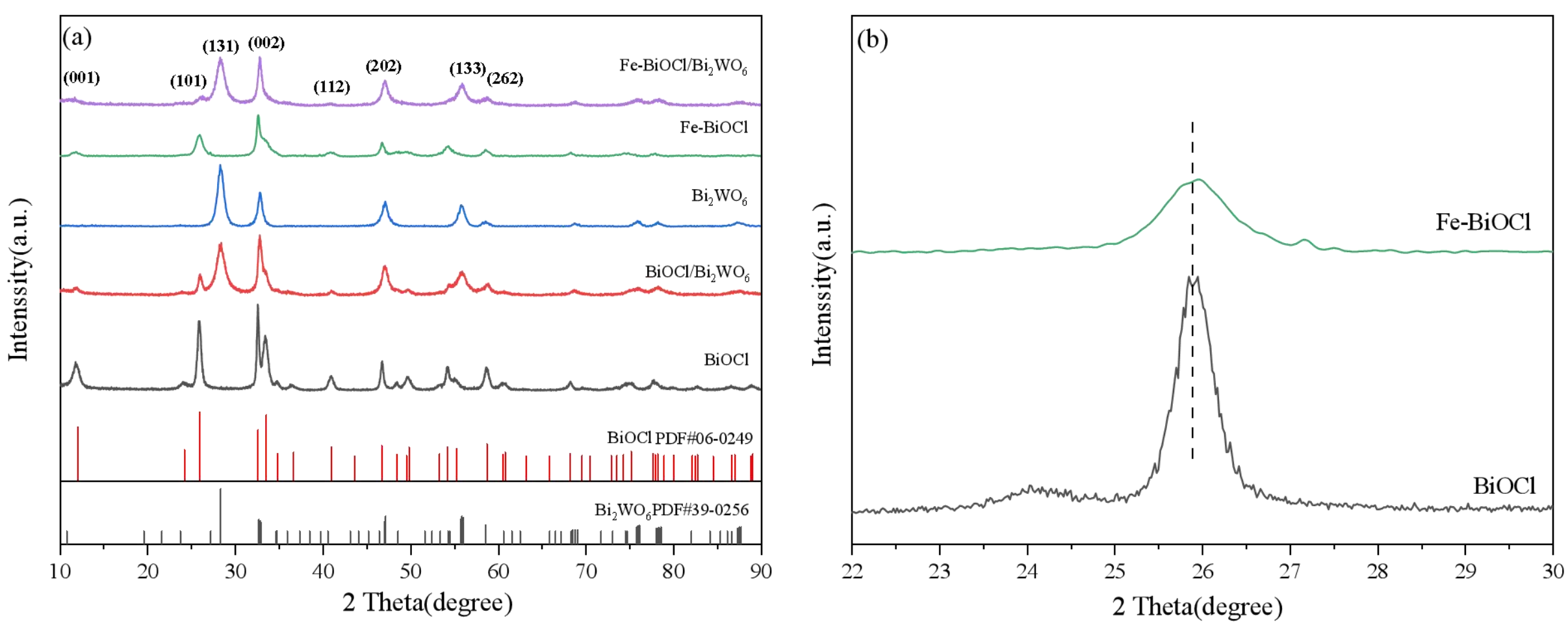

Fig.S2.(a)X-ray diffraction patterns of Fe-BiOCl/Bi_2WO_6, Bi_2WO_6, Fe-BiOCl, BiOCl and BiOCl/Bi_2WO_6 and (b)Enlarged view of parts of Fe-BiOCl and BiOCl

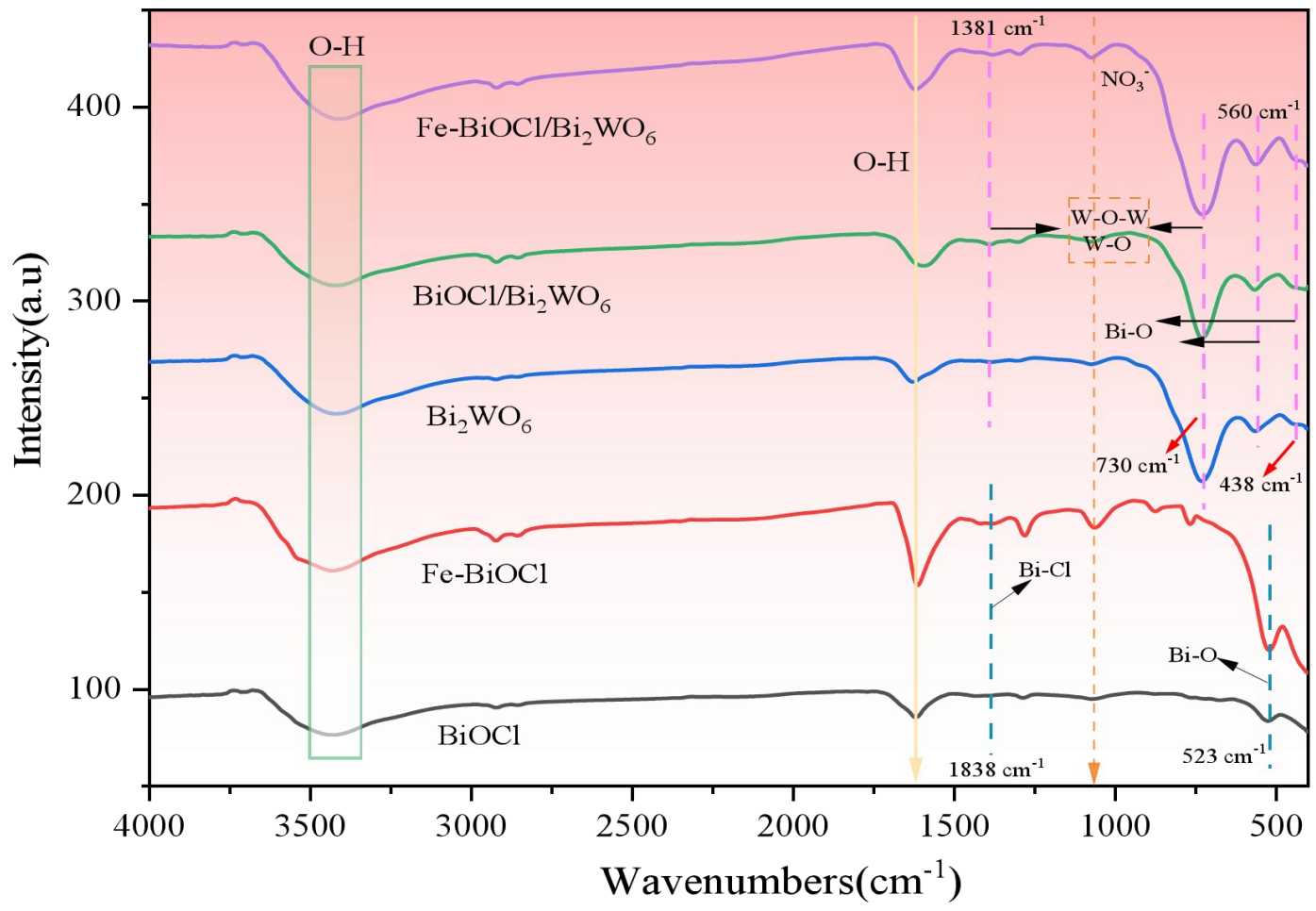

Fig.S3. Fourier infrared spectra of Fe-BiOCl/Bi_2WO_6, Bi_2WO_6, Fe-BiOCl, BiOCl and BiOCl/Bi_2WO_6

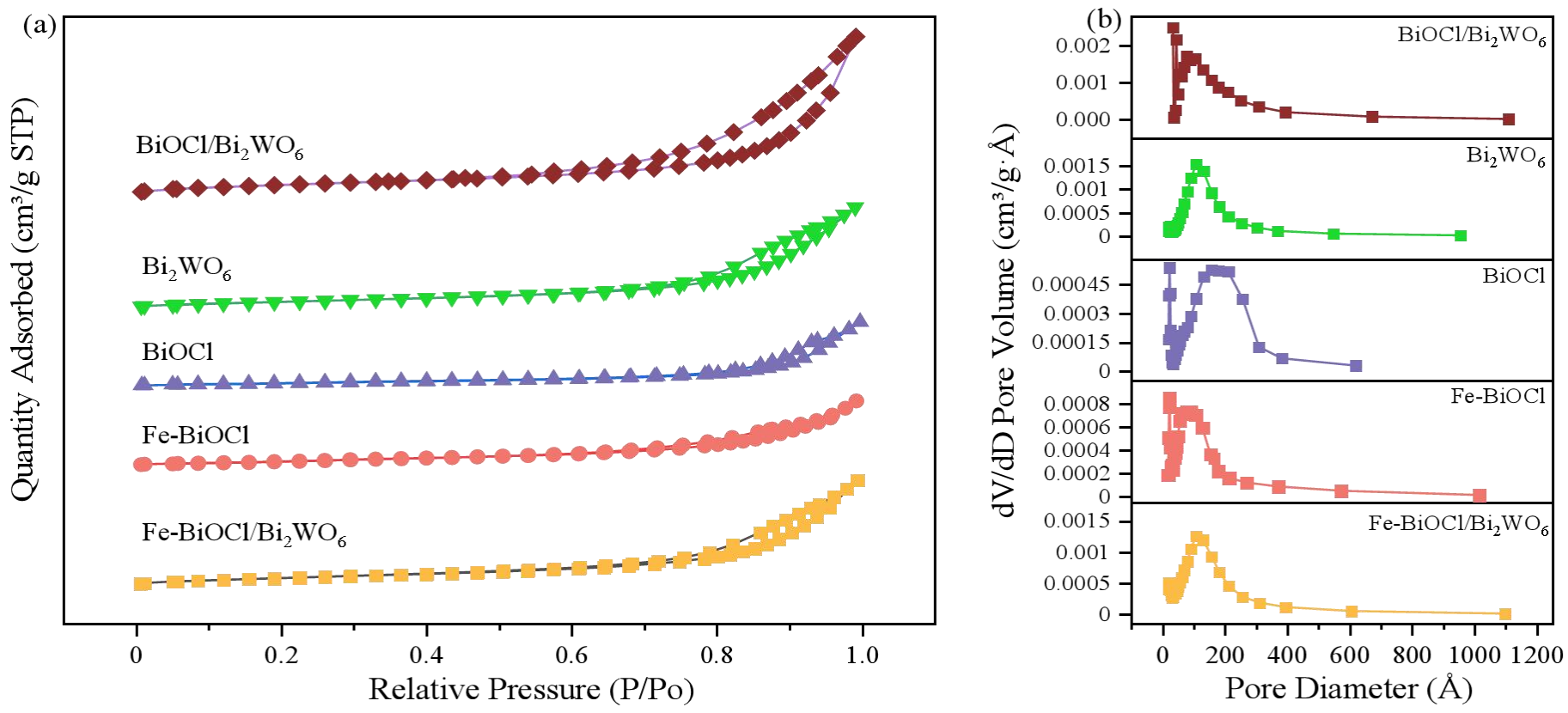

Fig.S4.(a) N_2 adsorption-desorption isotherm diagram and (b) pore size distributions diagram of Fe-BiOCl/Bi_2WO_6, Bi_2WO_6, Fe-BiOCl, BiOCl and BiOCl/Bi_2WO_6

Table.S1 BET data for Fe-BiOCl/Bi_2WO_6, Fe-BiOCl, BiOCl, Bi_2WO_6 and BiOCl/Bi_2WO_6

Material	Specific surface area （m^2/g）	Pore volume （cm^3/g）	Aperture（nm）
Fe-BiOCl/Bi_2WO_6	65.779	0.182	11.050
Fe-BiOCl	48.335	0.114	9.430
BiOCl	31.412	0.101	12.862
Bi_2WO_6	59.369	0.188	12.661
BiOCl/Bi_2WO_6	79.129	0.227	11.475

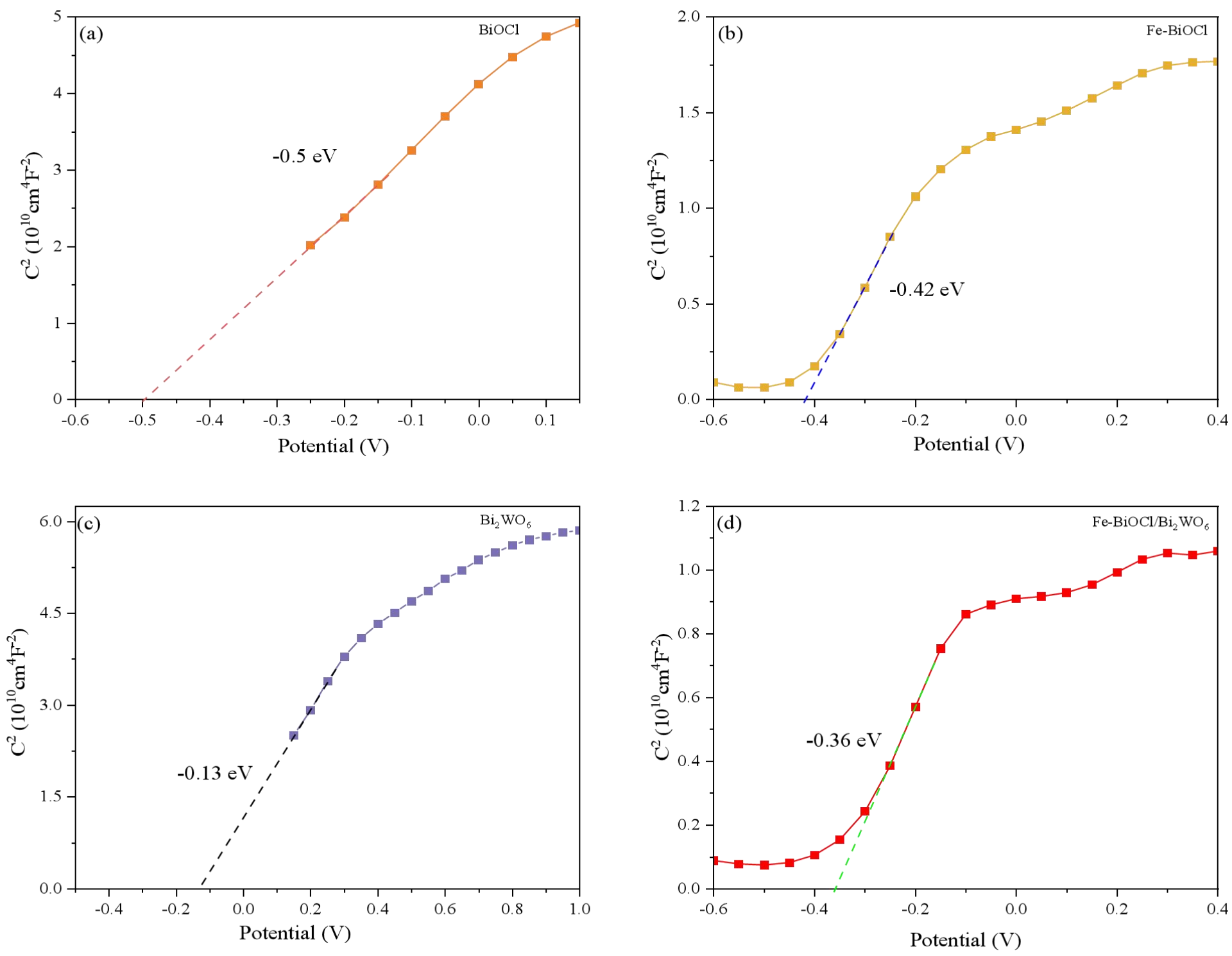

Fig.S5 Mott-Schottky plots of (a)BiOCl, (b)Fe-BiOCl, (c)Bi_2WO_6 and (d)Fe-BiOCl/Bi_2WO_6

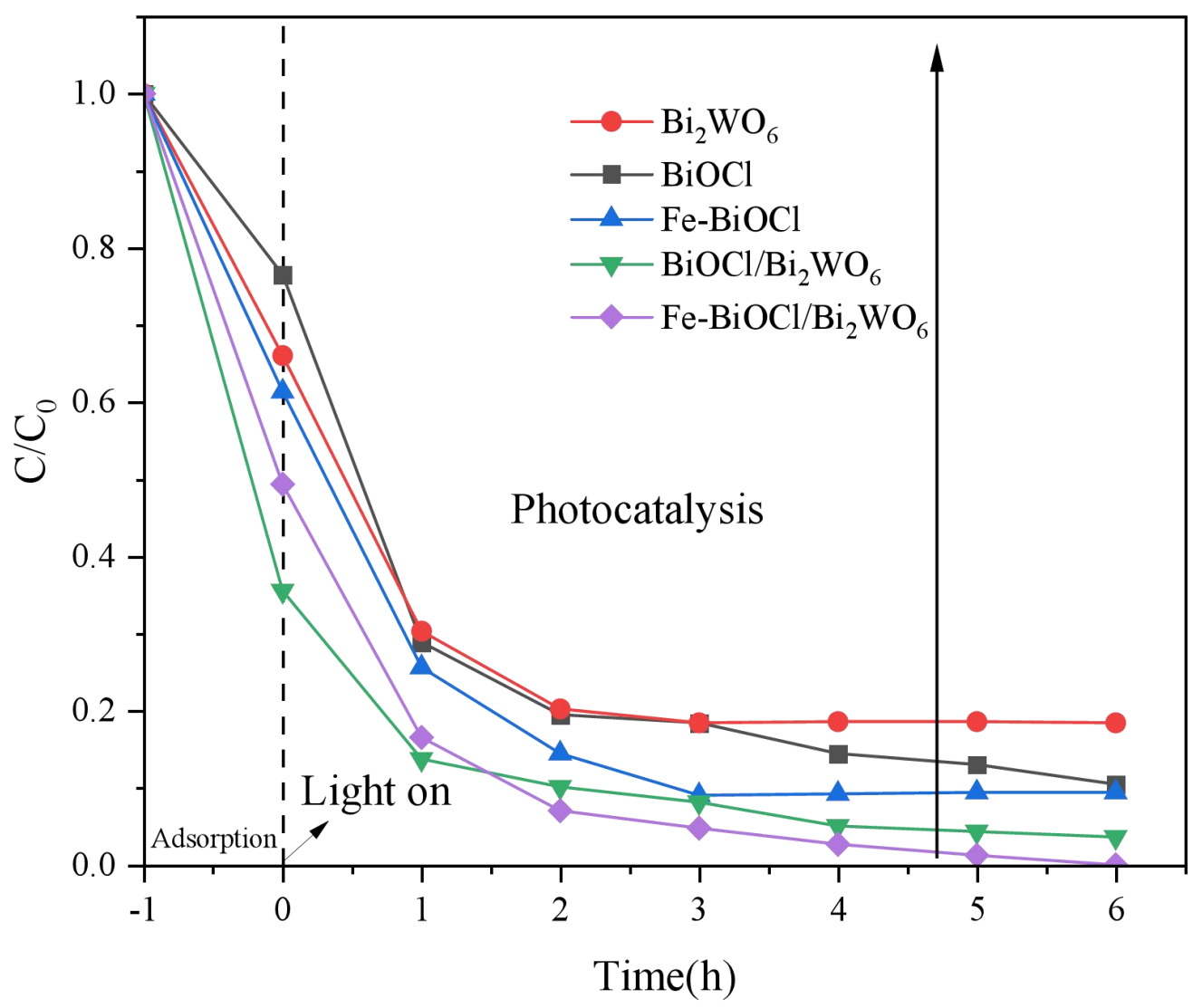

Fig.S6. Fe-BiOCl/Bi_2WO_6, Fe-BiOCl, BiOCl, Bi_2WO_6 and BiOCl/Bi_2WO_6 deoxidized LMF at 500 W visible light

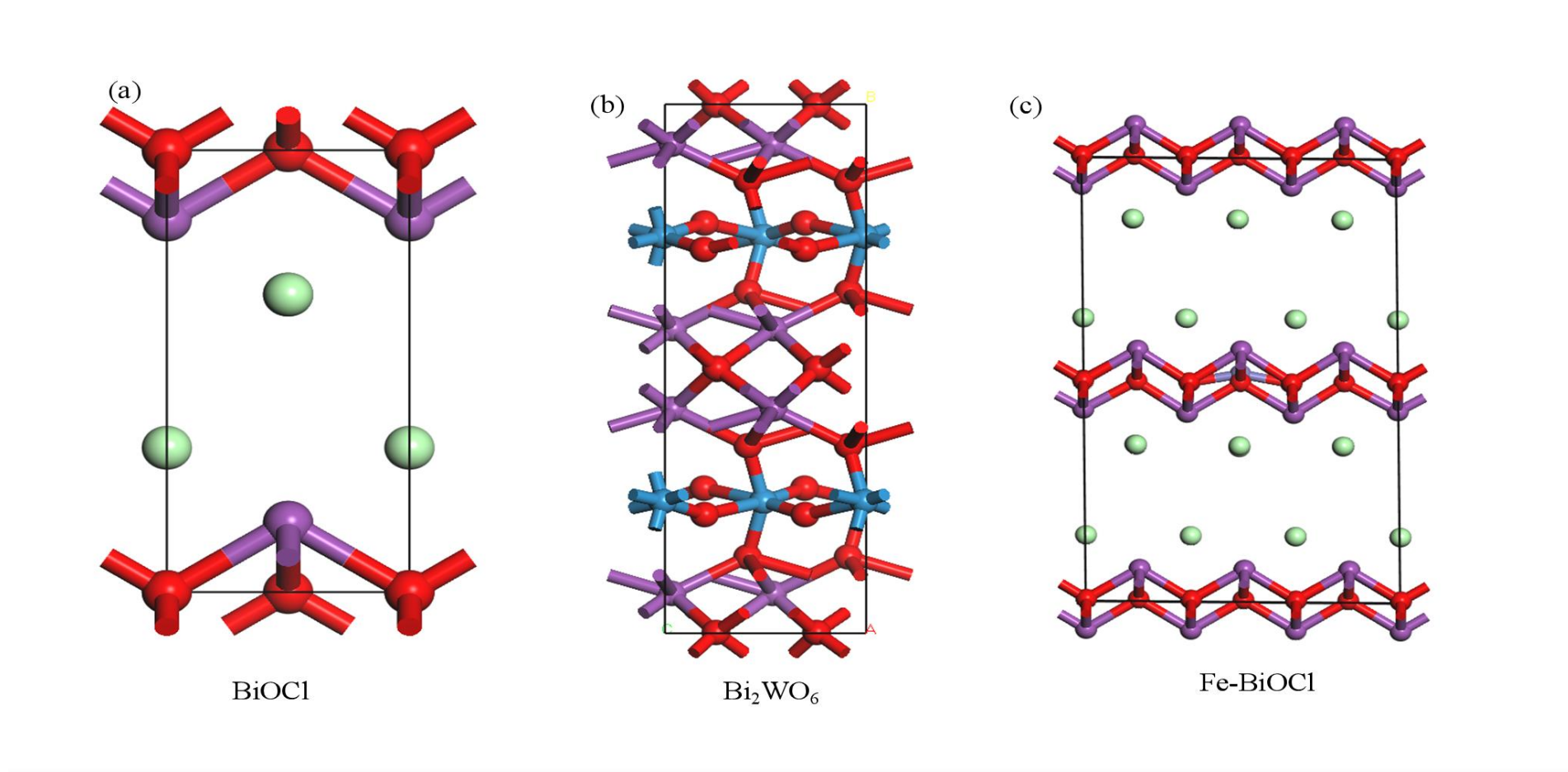

Fig.S7. Block model (a)BiOCl, (b)Bi_2WO_6, (c) Fe-BiOCl

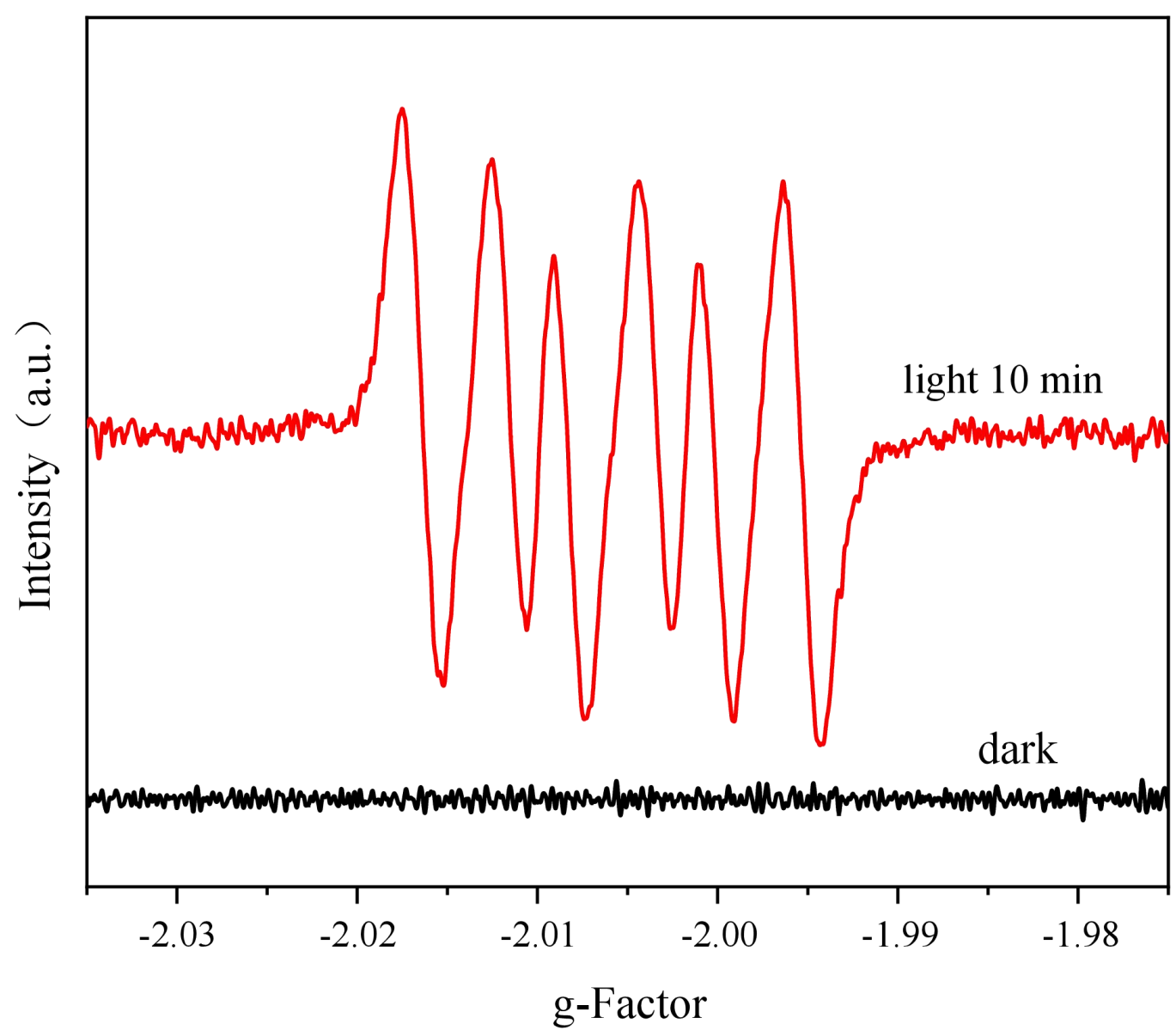

Fig.S8. EPR spectra at dark and after 10 minutes under visible-light irradiation of LMF degradation by Fe-BiOCl/Bi_2WO_6

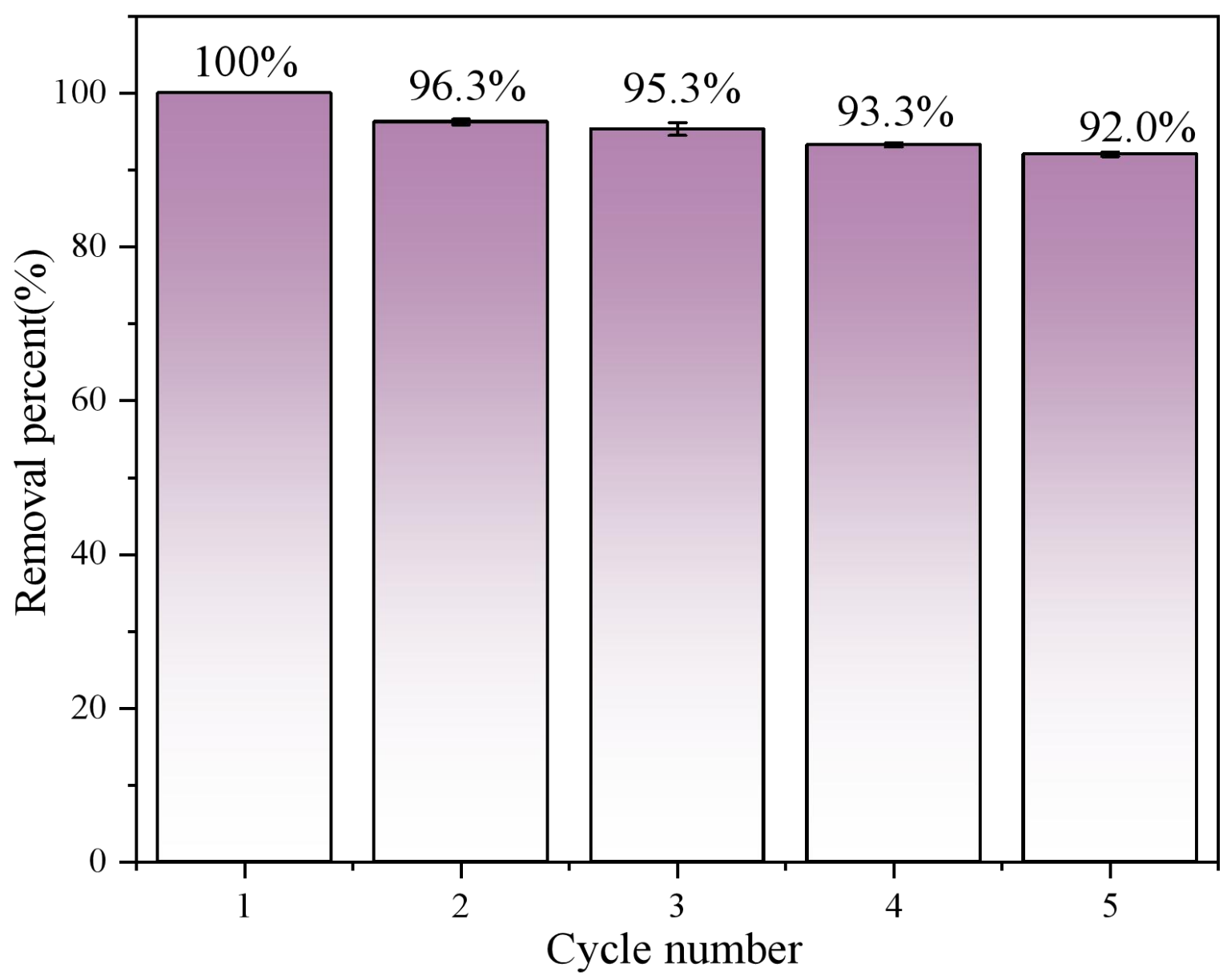

Fig.S9. The effect of cyclic Fe-BiOCl/Bi_2WO_6 photocatalytic degradation of LMF

Table.S2 DFT-calculated theoretical adsorption energy values

Molecule	Surface（eV）	Pollutant（eV）	Surface+pollutant up（eV）	Surface+pollutant down（eV）
LMF	-8.21903117E^{+4}	-6.38534706E^{+3}	-8.85751712E^{+4}	-8.85747601E^{+4}

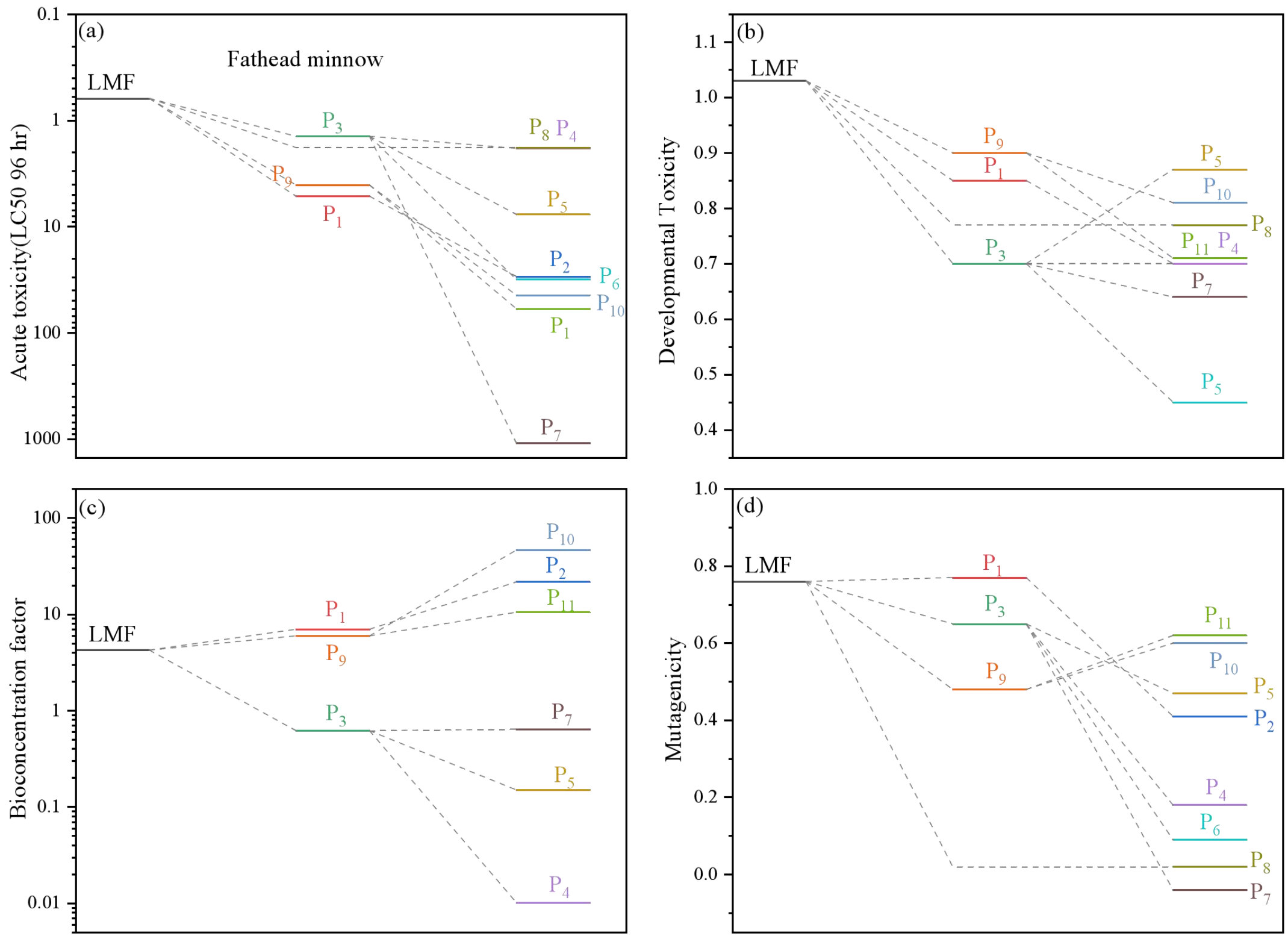

Fig.S10. Toxicity prediction of T.E.S.T. (a) Acute toxicity; (b) Developmental toxicity; (c) bioaccumulation factors; (d) Mutagenicity of LMF and degradation intermediates.

Table.S3. Leaching concentration of metal ions in solution after Fe-BiOCl/Bi_2WO_6 photocatalytic degradation

	Fe	Bi
LMF	19.05 μg/L	10.63 μg/L

Table.S4. Mineralization rate of LMF by photocatalytic degradation of each catalyst

	Bi_2WO_6	BiOCl	Fe-BiOCl	Fe-BiOCl/Bi_2WO_6
Mineralization rate (%)	20.36	24.75	31.08	47.43

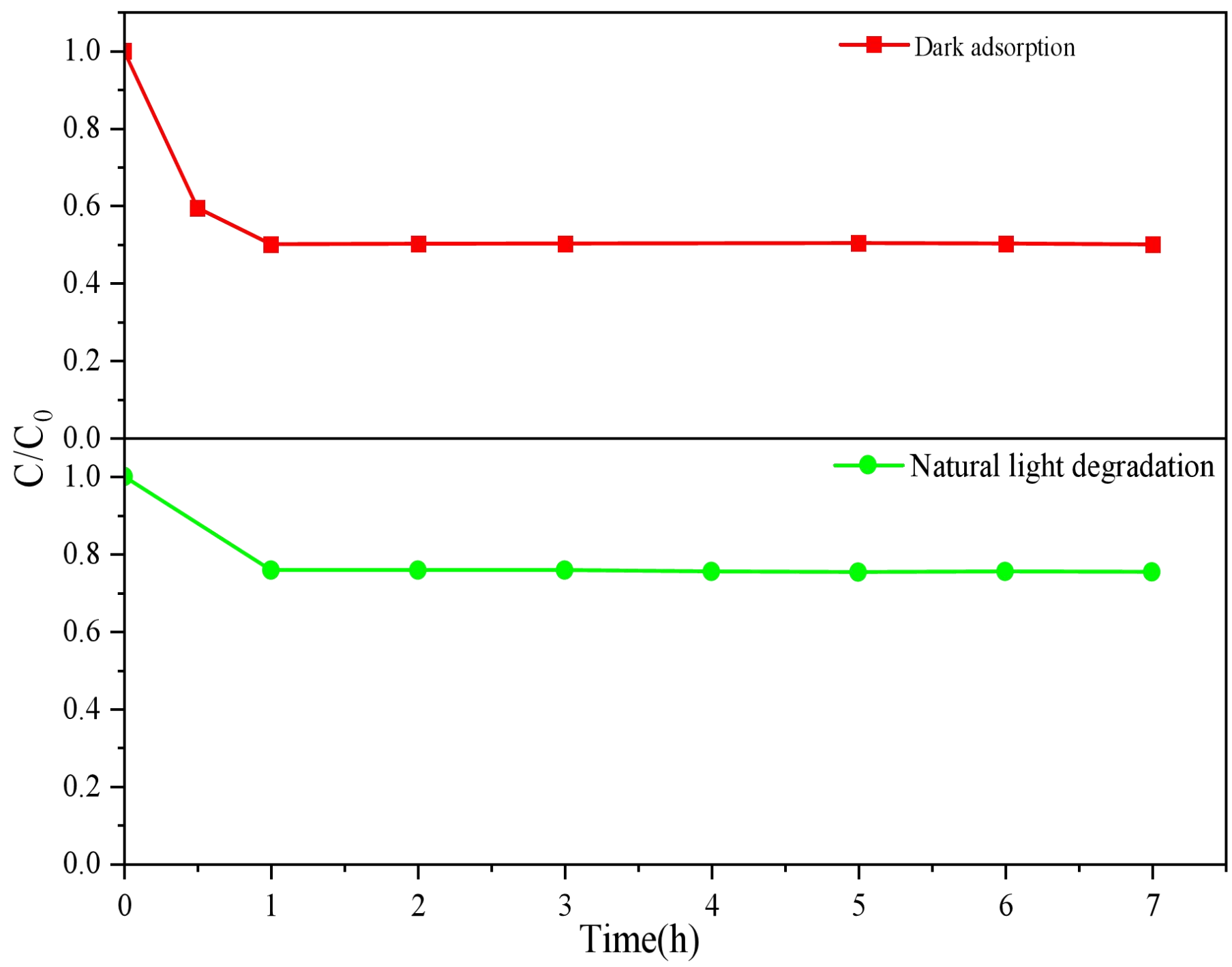

Fig.S11. Dark adsorption process of LMF on Fe-BiOCl/Bi_2WO_6 and (b) natural photolysis of LMF

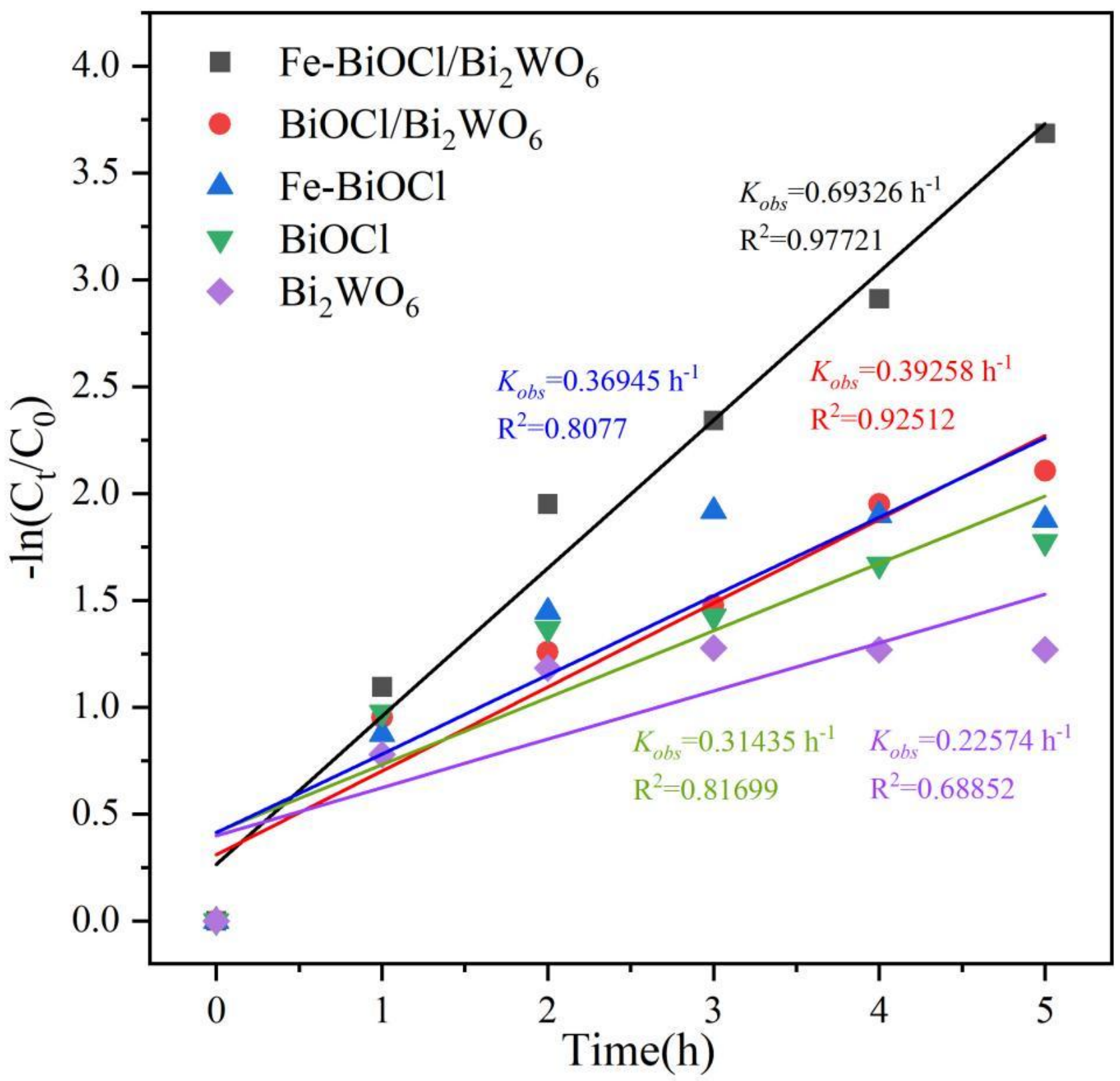

Fig.S12. Photocatalytic kinetic fitting results of LMF by Bi_2WO_6, BiOCl, Fe-BiOCl, BiOCl/Bi_2WO_6, and Fe-BiOCl/Bi_2WO_6 at 500 W visible light

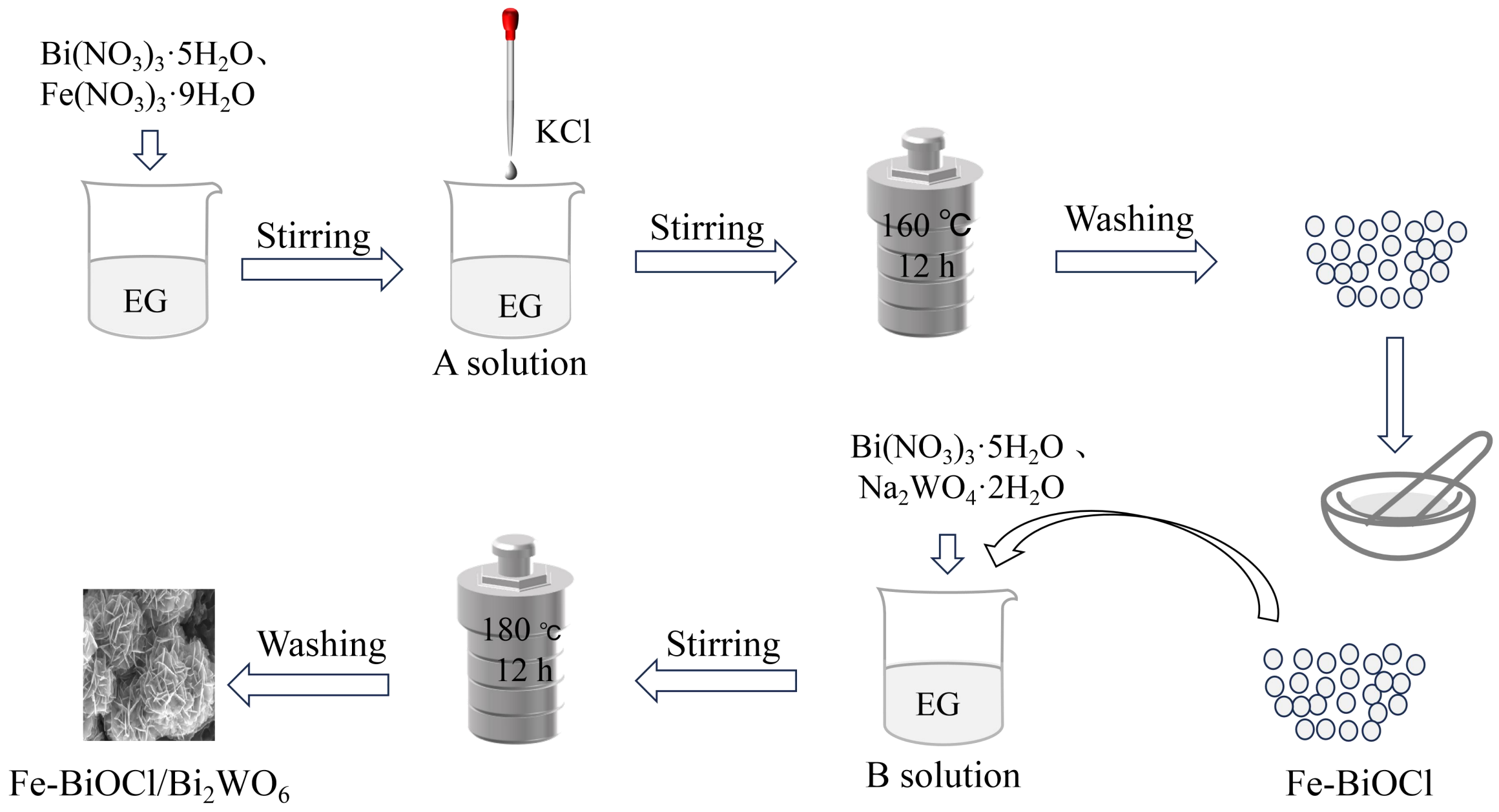

Fig.S13. Preparation process of composite catalyst
